# Guidelines on Asset Management of Offshore Facilities for Monitoring, Sustainable Maintenance, and Safety Practices

**DOI:** 10.3390/s22197270

**Published:** 2022-09-25

**Authors:** Chiemela Victor Amaechi, Ahmed Reda, Irish Mpho Kgosiemang, Idris Ahmed Ja’e, Abiodun Kolawole Oyetunji, Michael Ayodele Olukolajo, Ikechi Bright Igwe

**Affiliations:** 1 School of Engineering, Lancaster University, Lancaster LA1 4YR, UK; 2 Standards Organisation of Nigeria (SON), 52 Lome Crescent, Wuse Zone 7, Abuja 900287, Nigeria; 3 School of Civil and Mechanical Engineering, Curtin University, Bentley, WA 6102, Australia; 4 Department of Engineering, Qatar Energy, Doha 3212, Qatar; 5 Department of Business Management, University of Central Lancashire (UCLAN), Preston PR1 2HE, UK; 6 Department of Civil Engineering, Universiti Teknologi PETRONAS, Seri Iskander 32610, Malaysia; 7 Department of Civil Engineering, Ahmadu Bello University, Zaria 810107, Nigeria; 8 Lancaster Environment Centre (LEC), Lancaster University, Lancaster LA1 4YQ, UK; 9 Department of Estate Management, University of Benin, Benin City 300287, Nigeria; 10 Department of Estate Management, Federal University of Technology, Akure 340252, Nigeria; 11 Department Welding Engineering and Offshore Technology, National Centre for Nondestructive Testing, Petroleum Training Institute (PTI), Effurun 330102, Nigeria

**Keywords:** monitoring, offshore structure, oil and gas platform, asset management, health and safety, integrity management, risk assessment, life extension, audit, sustainability, safety practice

## Abstract

Recent activities in the oil and gas industry have shown an increasing need for monitoring engagements, such as in shipping, logistics, exploration, drilling, or production. Hence, there is a need to have asset management of these offshore assets (or facilities). Much of the offshore infrastructure is currently approaching or past its operational life expectancy. The study presents an overview on asset management of offshore facilities towards monitoring, safe practices, maintenance, and sustainability. This study outlines the major considerations and the steps to take when evaluating asset life extensions for an aging offshore structure (or asset). The design and construction of offshore structures require some materials that are used to make the structural units, such as offshore platform rigs, ships, and boats. Maintaining existing assets in the field and developing new platforms that are capable of extracting future oil and gas resources are the two key issues facing the offshore sector. This paper also discusses fault diagnosis using sensors in the offshore facilities. The ocean environment is constantly corrosive, and the production activities demand extremely high levels of safety and reliability. Due to the limited space and remote location of most offshore operations, producing cost-effective, efficient, and long-lasting equipment necessitates a high level of competence. This paper presents the guidelines on asset monitoring, sustainable maintenance, and safety practices for offshore structures. In this study, the management of offshore structures were also presented with some discussions on fault monitoring using sensors. It also proposes sustainable asset management approaches as guidelines that are advised, with policy implications.

## 1. Introduction

The continual challenge for facility managers and asset integrity managers is balancing asset design, maintenance, and replacement costs with the costs to the oil and gas business—in terms of finance, time, and resources—throughout their life-cycle [1,2,3,4,5,6,7,8]. It is, at its core, the management of asset depreciation, thus, there is a need for this research on sustainable asset management. Asset integrity management has evolved over the last few decades, from simple time-based inspections of key equipment to risk and reliability-centered management systems for all safety-critical parts [9,10,11,12,13,14]. Asset integrity management encompasses a number of elements that are critical to the long-term viability and serviceability of offshore installations, as seen in various field developments [15,16,17]. This would help to prevent offshore accidents like Ranger I Mobile Offshore Drilling Unit (MODU), Petrobras-36, Macondo Well’s Deepwater Horizon Blowout and Piper Alpha offshore accidents [18,19,20,21,22,23]. In general, the offshore sector includes various activities, including shipping, logistics, wind farm scheduling, facilities maintenance, exploration, drilling, and production [24,25,26,27,28,29,30,31,32]. Asset management has also evolved into different management systems. Asset management (or facilities management) has been identified as a key aspect of the oil and gas sector, which ensures longevity, life extension, serviceability, and aging assessment, among others, for these offshore facilities (or assets) [33,34,35,36,37,38,39,40,41]. However, recent issues that have challenged the offshore industry include the structural integrity of offshore assets [42,43,44,45,46,47]; offshore asset monitoring [48,49,50,51]; asset life extension [52,53,54]; risk assessments [55,56,57,58]; health, safety, and environment (HSE) [59,60,61,62,63]; monitoring indicators [64,65,66,67,68,69,70] and asset management [71,72,73,74,75]. With these systems running well, oil corporations and operators can achieve their goals of having sustainable oil exploration, and related activities. These offshore platforms must be well designed and maintained to avoid failure while being acted upon by different loadings. Hence structural integrity is necessary, especially on aging structures. Sensors are also applied onto various devices that are used to monitor offshore structures, and related applications that are used in offshore assets.

**Figure 1:**
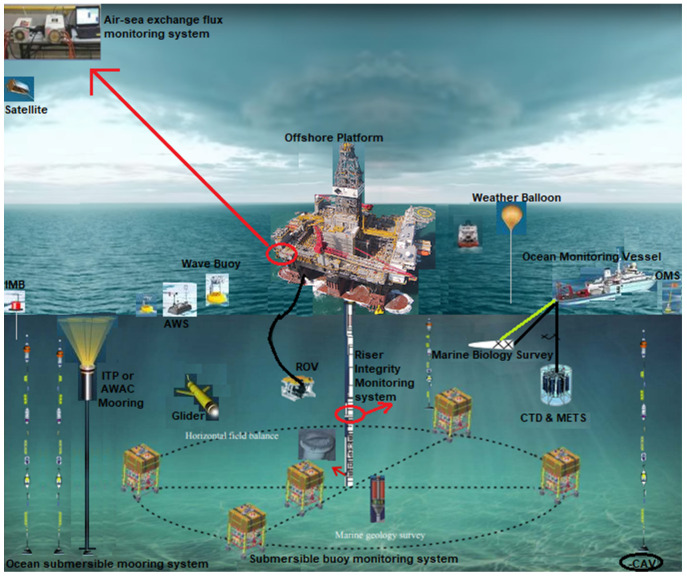
Different monitoring systems that are used on an offshore production asset, showing ocean submersible monitoring systems, submarine buoy monitoring systems, remotely-operated vehicle (ROV), conductivity-temperature-depth (CTD) instrument, air–sea exchange flux monitoring systems, ice mass-balance buoy (IMB), Ocean Monitoring System (OMS), weather balloon, satellite, etc. (Image Credit: Author 1-C.V.A.).

Facilities operations involve the activities that can be conducted to produce oil products, either onshore or offshore. Different factors also influence the type of asset management that will be considered as well as the metrics and indicators that will be used to evaluate the offshore facility [76,77,78,79,80,81,82,83]. These include the type of offshore platform [84,85,86,87,88,89,90,91], production operations [92,93,94,95,96,97,98,99], systems management model [100,101,102,103], fossil fuel deposits’ locations with historical exploration [104,105,106,107,108,109,110,111,112,113,114,115,116,117], etc. In the oil and gas industry, there are more challenges involving the application of offshore platforms in deep water locations [118,119,120,121,122,123,124,125]. These challenges include high water depths, harsh weather conditions, heavy windy conditions, and significant wave heights thus the need for risk assessments and facilities management [126,127,128,129,130,131,132]. However, there are elements that are factored into considering other aspects, such as project management, construction practices, site development, marine riser installation, deployment of floating offshore wind turbine (FOWT), and commissioning of offshore facilities, and riser monitoring [133,134,135,136,137,138]. Offshore structures are intended to be placed thousands of kilometres away from shorelines in the open sea, lakes, gulfs, and other bodies of water [139,140,141,142,143,144]. These offshore structures and their component attachments can be made from different construction materials, ranging from steel, composites, concrete, titanium alloys, and aluminium, to additive manufactured components [145,146,147,148,149,150,151,152,153]. Steel grades are used to construct most oil and gas platforms, hence the need for robust designs [154,155,156,157,158,159,160], skilled personnel for drilling/production activities [161,162,163,164,165,166,167,168,169,170,171], reliability analysis [172,173,174,175,176,177,178,179], and adequate corrosion control measures [180,181,182,183,184,185,186,187,188]. Offshore operations are conducted using industry standards like ISO, BS, NIS, API, ASTM, NORSOK, etc. Additionally, it is crucial that an accurate record of each offshore facility’s safety critical elements (SCE) and the condition of its equipment be kept. Figure 1 shows the typical monitoring systems that are applied on offshore production systems which are used to obtain data and monitor the offshore asset under production.

This study is conducted on the asset management of offshore facilities for structural integrity, safety, maintenance, and sustainability. Section 1 introduces the state-of-the-art asset management of offshore assets in the industry. Section 2 provides a general view of asset maintenance management. Section 3 presents sustainable facilities management while Section 4 discusses other parameters on asset management. Section 5 presents policy implications while concluding remarks are given in Section 6. Recommendations are offered for high longevity, high serviceability, long-term operations, and sustainability. This paper presents some guidelines with an overview on asset management, and also discusses fault diagnosis using sensors in the offshore facilities. Some standards that are useful with related patents are also presented. Lastly, the challenges of managing offshore structures using asset management systems towards sustainable maintenance and safety practices are also presented.

## 2. Asset Maintenance Management

The development and design of floating and fixed platforms are based on some design criteria. All operating considerations and environmental data that potentially affect the platform’s detailed design are included in the design parameters that are discussed here.

### 2.1. Asset Management

Asset Management (AM) is described as “the coordinated activity of an organization to realize value from assets” [9] in the ISO 55000 standard. AM tactics should be in line with different organizational strategy levels (corporate, business, and functional-level strategy). Due to intense worldwide rivalry, unpredictability, volatility, and insecurity, asset management has grown increasingly difficult and demanding. These organizations engage with considerable uncertainty and terrifying hazards of many kinds. These are the key strategic, operational, financial, organizational, and technical issues that have a significant impact on all of the company’s business operations. As a result, organizations face significant pressure to cut back on dangerous and unexpected equipment failures, which raises the bar for maintenance.

**Figure 2:**
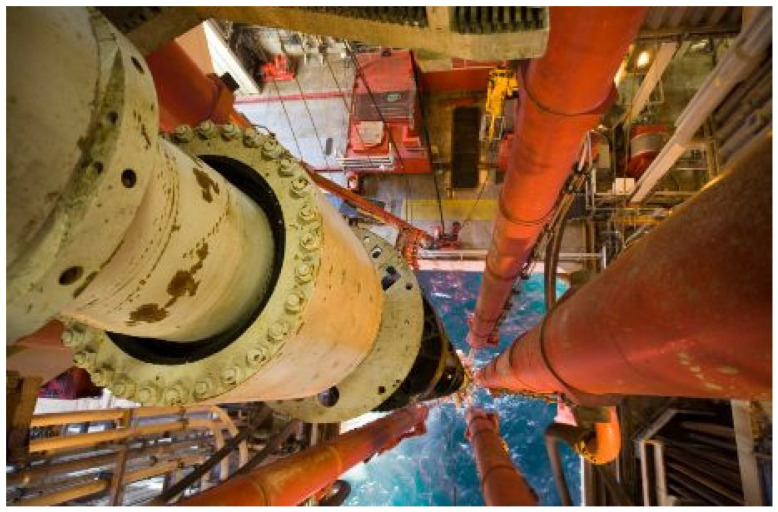
An aging offshore platform showing the marine riser.

Facility managers require proficient skills in facilities management for maintaining assets. On the other hand, engineers and project managers require integrity management skills. These skills are used with relevant strategies to make sure their operations are safe because oil and gas assets are constantly under strain. Training and leadership are key elements of asset management, as the offshore industry is constantly undergoing training of personnel in different units and at various levels. Sometimes, consultants are used for expert opinions, training, and consultations on relevant aspects of the assets to be managed. Staff need to be trained despite having prior training, which is called on-the-job training. Also, the project leader should consult or seek practical advice when required. Engineers may prepare and reduce safety risk by using the essential skills on asset integrity management of offshore structures [9,10,11,12,13,14]. However, more attention is required on aging structures and structures that are under life extensions or are undergoing structural integrity assessments. Sensors are also used to access the integrity and monitor different devices that are applied on offshore structures [109,171], such as marine risers, as seen in Figure 2.

### 2.2. Accident Reporting

**Figure 3:**
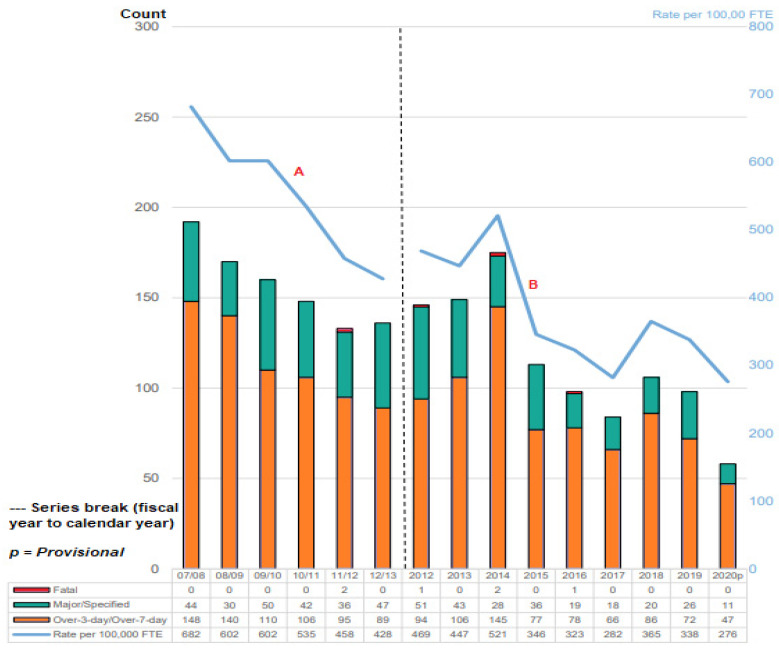
Oil and gas offshore injury rate showing fatal injuries, over 3 days/over 7 days, major injuries, and rate per 100,000 FTE for all reported offshore injuries, from 2007/08 to 2020 using RIDDOR database for full time equivalent (FTE) workers. Note: A and B show two different accident reporting styles by years, such as 12/13 and 2014. (This image is re-used/reproduced with permission of the Health and Safety Executive under the terms of the Open Government License, Courtesy: HSE, UK. Source: RIDDOR and HSE [190]).

In the UK, accidents in workplaces have to be reported via an HSE reporting system which then gets recorded into an accident report database. One of such databases is the RIDDOR database. Accidents are reported, despite the type of accident, level of accident, or the age of the structures, as illustrated in Figure 3. The RIDDOR covers various workplace hazards, particularly the offshore industry. There are also dedicated databases for failure cases of offshore assets such as pipelines and marine risers. Hence, aged offshore structures also have some tendency to cause accidents, such as corroded pipelines, clogged valves, etc. However, since some of these platforms have aged, it has become more important than ever to ensure their sustainability. Also, some of these long-standing offshore facilities need to be maintained, monitored, and accessed at different times to control structural failures due to aging, lack of proper checks, and the need for some repairs. Also, the health and safety of personnel must also be factored. Different HSE reports show that fewer injuries occur each year on offshore oil and gas platforms [189,190], such as the plot in Figure 3.

Based on the Occupational Safety and Health Administration (OSHA) method, the analysis by HSE and Hazardex [190,191,192,193,194] assumed that a full-time equivalent works 2000 h year. RIDDOR data are based on fiscal year (April–March) for the years 2007/08 to 2012/13, while the data starting in 2012 to 2020 is based on the calendar year (January–December). Additionally, recent changes to the regulatory requirements and reporting structure make it challenging to compare current data with earlier periods. Key findings for the reported injuries in different years, such as for 2018 which included a total of 106 injuries that were recorded under RIDDOR, with a rate of 365 injuries per 100,000 FTE and no fatalities (see Figure 3). Despite the fact that there have been six fatalities in the past ten years and three in the previous five, 2018 marked the second year in a row without a fatality, as well as 2019 and 2020. According to estimates using data of Persons on Board (PoB), there were 29,000 full-time equivalent (FTE) workers offshore in 2018, up from 29,700 in 2017 and 30,400 in 2016. FTE is based on the idea that an FTE employee works 2000 h a year and that each shift lasts, on average, 12 h, thus Equation (1):

FTE = 2000 × Total PoB Nights × 12(1)

Similar to the other two years covering 2016 and 2017, 19 specific injuries were documented. In contrast to 61 per 100,000 FTE in 2017 and 63 per 100,000 FTE in 2016, the rate was 66 per 100,000 FTE workers in 2018. However, in 2019 there were 98 injuries that were reported via RIDDOR, at the rate of 338 injuries per 100,000 FTE workers. However, the legal requirement to report workplace injuries that resulted in more than three days of absence (often known as “over-3-day”) changed to “over-7-day” in April 2012 [195].

The main themes for the 2018 data were 87 injuries over-7-day injuries were reported, while up from 66 in 2017 and 98 injuries in 2019. Also, there were 338 injuries per 100,000 FTE in 2019, while there were 300 injuries per 100,000 FTE in 2018 and there were 222 injuries per 100,000 FTE in 2017. Also, the HSE study reports [190,191,192,193,194] showed that there were different types of injuries that were reported in 2012–2020. In 2018, there were fractures (represented roughly 90% of the reported specific injuries) (17 of 19), sprains, and strains (which made up 32% of reported injuries lasting more than 7 days) (28 of 87). Also, the 2018 data showed that out of all the reported injuries, 50% involved the upper limb (53 of 106) while lower limb injuries made for 26% of all the reported injuries (28 of 106). In total, limb injuries in 2018 accounted for all 19 of the listed injuries and 71% of injuries lasting longer than seven days (62 of 87). In 2019, there were 73 over 7-day injuries, where the rate of injuries per 100,000 FTE workers was 252, compared to the 2018 data which had 86 over 7-day injuries, with the rate of injuries per 100,000 FTE workers of 296 [190,191,192,193,194]. In 2020, provisional data were utilized (see Figure 3 shown as 2020p), as it had 58 injuries that were reported via RIDDOR, at a rate of injuries per 100,000 FTE workers of 276, and had the least recorded injuries based on the accident reports made at 11 injuries, provisionally. In 2020, there were 52 injuries per 100,000 FTE workers, in comparison to the 90 injuries per 100,000 FTE workers in 2019; while 2020 had 47 over-7-day injuries, in comparison to 72 over-7-day injuries in 2019 [190,191,192,193,194].

### 2.3. Asset Life Extension (ALE)

Since the structures from both offshore marine and energy sectors operate in comparable environmental conditions, the consideration in post-design life scenarios might readily be applied to offshore wind. While the majority of the offshore wind farms that are already in place throughout the world have not yet completed 15 years of operation, it is still a goal to the hardware that is more than 50 years old in engineering structures that are on the verge of becoming obsolete [196,197,198,199,200,201,202,203,204,205,206].

Approximately one-third of all the active platforms in the North Sea are currently older than 25 years. The Aging and Life Extension Network, a group of 90 members that includes operators, ICPs, designers, contractors, and HSE, has allowed them to retain this quantity [189]. The objectives for conducting such ALE include to exchange information that are helpful on aging techniques, updating relevant practices, pinpointing crucial aspects of the aging process, analyzing accident reports [189,190,191,192,193,194,195], and creating guidelines.

**Figure 4:**
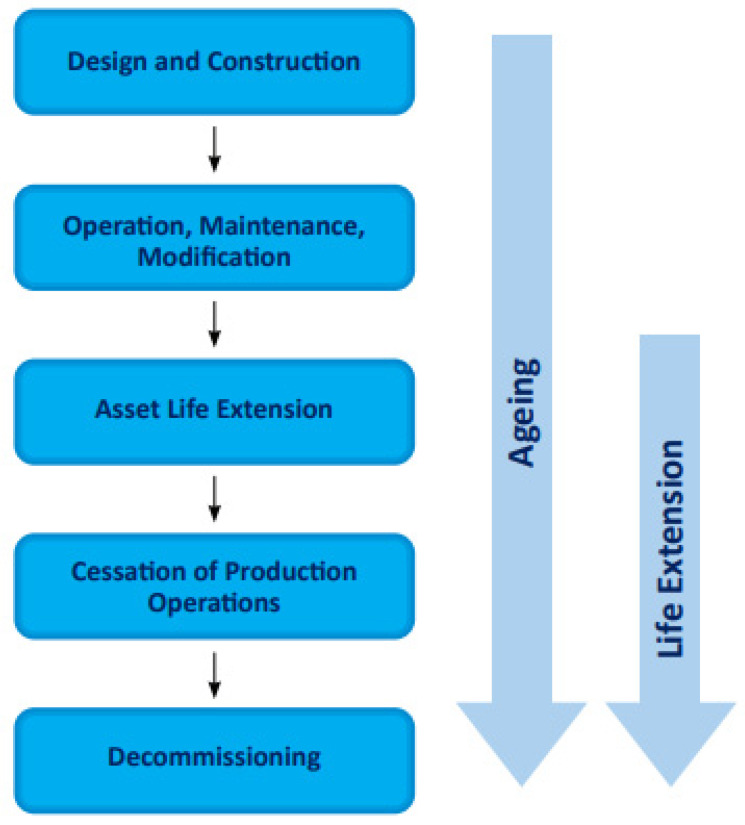
An illustration of the aging life cycle. (This image is re-used/reproduced with permission of the Oil and Gas UK. Publishers: OGUK & OEUK, Copyright year: 2012, Source: [209]).

Assets must be able to forecast and comprehend the consequences of degradation or possible changes that are related to life extension, as well as be ready to respond ensuring that demand is met without jeopardizing asset integrity and safety. For a certain design life expiry, asset life extension (ALE) refers to a condition in which an asset is approaching its intended design life. Material degradation, obsolescence, and organizational difficulties are the key aging variables to consider when creating an ALE program. Some industry standards and guidelines on asset management include OGUK guidelines [207,208,209], EI guidelines [210,211,212,213,214,215], DNV standard [216], NORSOK standard [217], ISO standard [218,219,220], and HSE guidelines [189,221,222,223,224,225] present different protocols for aging structures and conducting ALE assessments on offshore assets. Some of the advances that have been made on the aging protocols include the Key Programme 4 (KP4) initiative in 2010 [222] and HSE report RR509 in 2006 [223]. An illustration of the aging life cycle is represented in Figure 4.

The current state of known degradation mechanisms that can be applied to safety barriers should be assessed and documented. As a justification for the new mode and timeframe for continuous operations, the premise for deviation acceptance and management of change (MoC) is examined. All the changes must be evaluated by engineers, and finally mitigation methods against all operating risks must be documented. For the life extension period, oil and gas producers (OGPs) and subject matter experts (SMEs) must study, evaluate, and assess all the damage processes or faults that may affect the facilities or individual operating systems. This is often applicable to damage or faults for which a temporary MoC was allowed owing to a limited time of use that was later amended due to ALE concerns. After that, the OGP must re-evaluate the grounds for acceptance to ensure that it is still acceptable for the next time. Components or systems having a high risk of failure and that cannot be inspected must be discovered, evaluated, analyzed, and qualified for life extension. OGPs must assess the consequences of failure, monitor failure indicators, and have strategies in place for compensatory actions if failure indicators are discovered [129,130].

**Figure 5:**
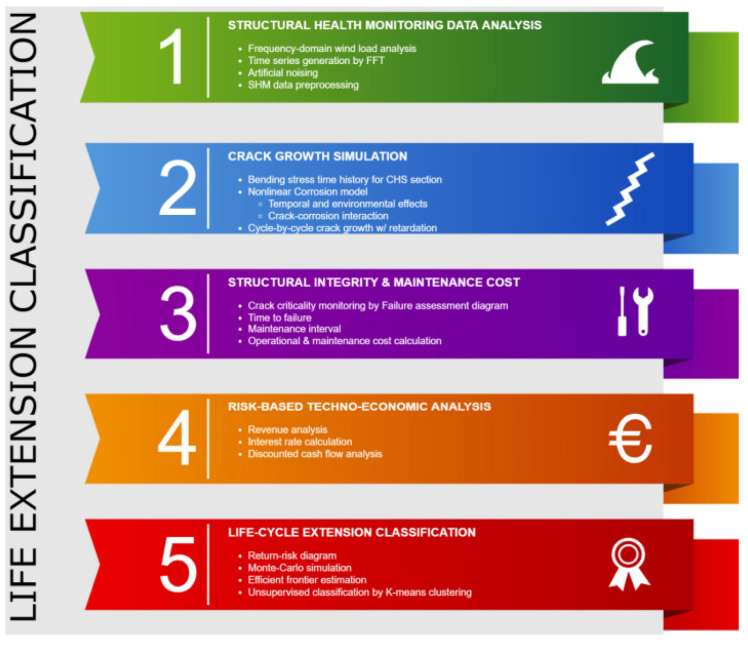
Flow chart for developing a typical life extension classification for an offshore wind farm. (Permission to use image is obtained from the authors—Baran Yeter and Yordan Garbatov, and used under MDPI open access rules. Publisher: MDPI, Copyright year: 2021, source: [196]).

OGPs should provide the following information in their ALE Study Consent for Extension Report at a base level:

- A clear understanding of how the asset will be used during the extension period.
- There should be a well-defined route in preparing the economic analysis.
- Clarity on the asset’s fitness for service as it approaches design life, remnant life assessment, life extension requirement, and gap closure requirement.
- Life extension classification can be prepared based on the type of study. For instance, a typical life extension classification for an offshore wind farm is seen in Figure 5.

### 2.4. Risk Assessment

The phrase “risk assessment” refers to a broad range of methods that are used to evaluate the degree of safety by factoring both the potential for harm and its likelihood. These evaluations may be qualitative, quantitative, or a combination of the two. Therefore, a risk assessment will include some evaluation of the damage and an evaluation of the likelihood. Such evaluations might be provided using straightforward qualitative scales or fully quantified to produce a risk value in numbers. A quantitative risk assessment (QRA) can use a variety of techniques or models, from straightforward correlations to intricate computer codes. These will include techniques for evaluating the effects of releases, such as dispersion models, flame radiation models, explosion models, models for evaluating the impact or damage that is caused by the fire, explosion, or impact. Risk assessment also provides information on the likelihood of failures of various pieces of equipment, and related applications, such as, on offshore assets [205,206,207,208,209,210,211,212,213,214,215].

A complex system (such as a platform or chemical plant) that includes hazardous materials, hardware, control and safety systems, personnel, and management systems is analyzed using QRA methodologies in order to ascertain the following the type of mishaps that are possible—depending on the study’s scope, the frequency of the accident’s occurrence and other factors that are found when it is created utilizing checklists, HAZOP, or prior research knowledge. Also, based on the frequency of different accidents happening, a fusion of conditional probabilities and statistics on failure rates are also useful in informing decisions.

Risk assessments must be carried out to ensure that the facility’s risk level remains below acceptable levels over the life extension term, and that it is as low as reasonably practicable (ALARP). The ALARP principle is widely used in the oil and gas industry. Based on the context that is established for life extension, the following risk assessments will be carried out:

- Amassing of operational risk assessments (ORA), which are sometimes decoupled since they were considered separately rather than in tandem, possibly resulting in unforeseen elevated hazards.
- Risk evaluation of significant disaster risk
- Quantitative/qualitative risk analysis (QRA)
- Occupational safety, health, and working conditions
- External environments
- Response and emergency preparations.

Assuring that risks have been lowered to ALARP levels necessitates balancing risks against costs in order to further reduce them. Since OGPs are expected to apply the risk reduction strategy, the decision is skewed in favor of health and safety. However, it is intended that the most up-to-date technology and knowledge in the field of risk assessment would be used for the analysis of any major accident. For all continuous operations, the level of conservatism and any assumptions that are made in risk assessments must be analyzed and evaluated. The risk assessment must encompass the vulnerability, current, and predicted effectiveness of the barrier function, as well as technological, organizational, and operational components [129,130]. Although, a consequence axis and a likelihood axis make up the OGP risk matrix, as shown in Table 1 for a typical risk register. The repercussions are plausible possibilities that can arise from the discharge of a hazard (taking into account the current circumstances). The worst-case scenarios, rather than the actual ones (that may have occurred) should be considered in the risk matrix.

### 2.5. Gap Assessment

Another stage of the ALE process is gap assessment. Gaps can be identified in a number of ways, including:

- Identifying risks and key barriers.
- Inspect the integrity and operation of the barriers.
- Evaluate the barrier’s present performance in terms of intent.
- Examine the performance of obstacles in the past.
- Examine the present condition of maintenance and identify any gaps.

The barrier functions and the factors that influence the barrier elements will be the subject of the gap/needs assessment. Gap assessments factor in operational, organizational, and technical factors. The recommendations of this assessments are made based on root cause failure analysis reports, reliability data, major inspection findings, overhaul observations and results, maintenance principles, maintenance reports, equipment modifications, list of faults, incident histories, operational philosophies, and recommendations for condition monitoring. Most suggestions for extending the life of a product must take into account the product’s future technical state, operating conditions, and mode of operation. The evaluation should also involve a study of the expected production profile, taking advantage of synergies with other relevant equipment to rationalize, optimize, or increase essential assets and system infrastructure.

**Figure 6:**
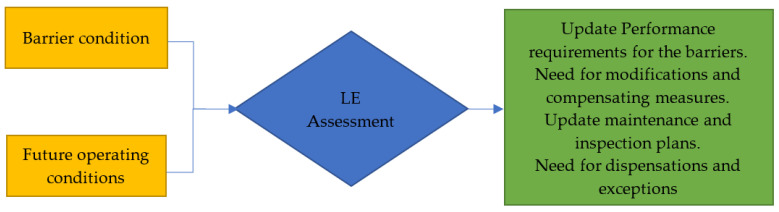
Recommended life extension assessments of barriers.

The gap assessment’s recommendations are to cover all of the necessary corrections. The activities required to avoid failure on offshore platforms, the risks involved handling and use of offshore equipment must be taken into consideration. These risks include obsolescence, failure modes prediction, replacement strategy, equipment delivery, spare part/replacement part, remnant life analysis, and the prediction of fatigue degradation mechanisms, particularly during the extended period. The advantages of using new technologies to close the gaps must be assessed. This may make it easier to reduce or close gaps with fewer adjustments or compensatory methods. The most up-to-date knowledge on degradation and life extension should be used [129,130], as seen in recommended life extension assessments of barriers, depicted in Figure 6.

### 2.6. Emergency Response System

On offshore assets, emergency preparedness is a crucial step to take. A review of the present emergency response systems is required, as well as an assessment of how operational changes and additional needs were addressed during the life extension term of a plant based on the HSE Case that was examined after changes to the operating philosophy [208,209,210,211,212,213,214,215]. Any potential operational or organizational changes to the facilities that will affect the emergency preparedness and response systems need to be evaluated by OGPs [129,130].

Due to the oil industry’s complexity, oil companies must be ready to respond to a variety of potential disruptions, including minor mishaps, oil price swings, political instability, epidemics/pandemics, severe accidents, and harsh weather. A key goal should be to prevent incidents through solid project planning, design, implementation, and leadership. However, if an oil leak or other unanticipated catastrophe occurs, procedures and processes should be in place to successfully respond. These oil companies should also conduct thorough investigations into all major occurrences to determine the core cause and share lessons that are learned in order to avoid such incidents in the future. Annual reports on spill performance should also be provided.

However, to prevent accidents and control hazards on offshore installations, sufficient preparedness policies should be in place. A Crisis and Emergency Management Plan should be in place as well, laying out the structure for dealing with major occurrences of any kind. Should a crisis occur, a Crisis Communications Functional Support Plan which lays out how these companies will communicate with internal and external stakeholders, should be implemented. This crisis support plan covers aspects of emergency responders, community members, regulatory agencies, and family members. Each division should keep emergency response plans that are tailored to the hazards that each asset poses. All workers, contractors, and designated suppliers have access to the response plans.

In an emergency, a thorough tiered response system might be used to quickly assemble the relevant teams. ConocoPhillips’ tier system, for example, is employed during emergencies [10,24,25,26,27,28]. At the business unit level, a Tier 1 response is fully managed. As part of our Tier 2 and Tier 3 response frameworks, if the reaction exceeds the capacity of a single business unit, the Crisis Management Support Team and Global Incident Management Assist Team (GIMAT) will be activated. During a major incident or crisis, the Crisis Management Support Team provides functional, strategic, and/or tactical support to the afflicted business unit. The GIMAT is made up of company-wide subject matter specialists who have undergone comprehensive emergency response training. The Crisis Manager would give direct access and updates to the Executive Leadership Team in a Tier 3 response scenario.

### 2.7. Aging Management/Obsolescence

**Figure 7:**
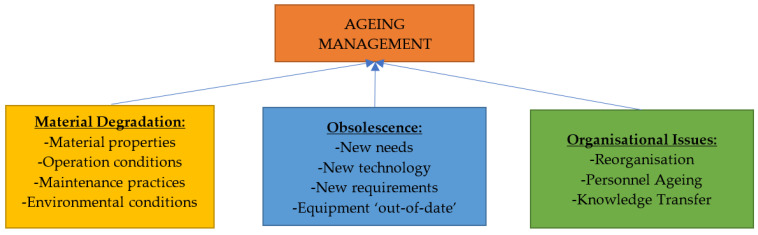
Aging Management.

On offshore facilities, aging management is conducted to ensure that the status of obsolescence is up-to-date, and necessary maintenance management systems that are relevant to the asset that is under study. Obsolescence study is conducted to obtain the status of different operations of the offshore facility, to ensure that compliance was met in accordance with relevant international standards and industry regulations. Aging management is classified to three (3) areas, namely the material degradation, obsolescence, and organizational issues, as illustrated in Figure 7.

**Figure 8:**
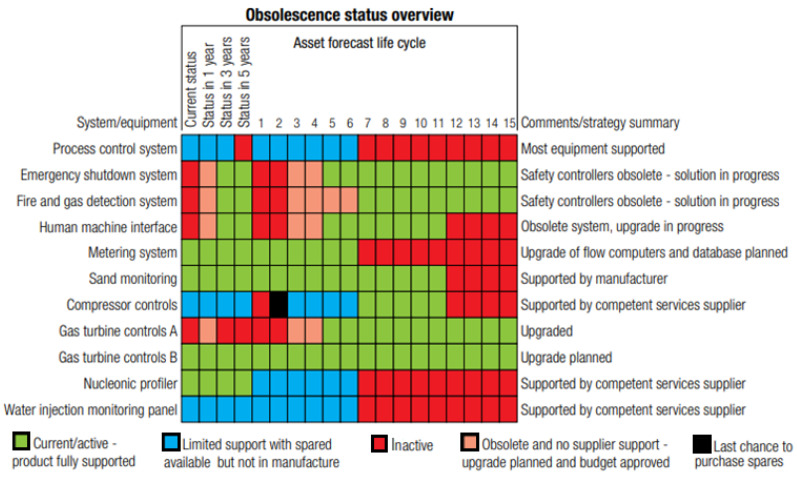
Example of an obsolescence status overview chart, showing different aspects for the lifecycle of an offshore asset (This image was adapted with permission of the Health and Safety Executive under the terms of the Open Government License, Courtesy: HSE, UK. Source: [189]).

Asset integrity and reliability are dependent on effective inspection and maintenance. An initial analysis is essential when creating maintenance management systems to determine the status and how the aging processes are covered in the existing maintenance program. Using the aging management protocol that was developed in Figure 7, there is a need to update the status of each operation, and the related systems based on integrity, dependability, vulnerability, and consequence analysis for future continuous operations [189,222]. The evaluation must include experience and knowledge that is gained from documented failures and lessons that have been learned, which will be used to improve the maintenance management system. A typical chart of the obsolescence status overview is given in Figure 8.

**Figure 9:**
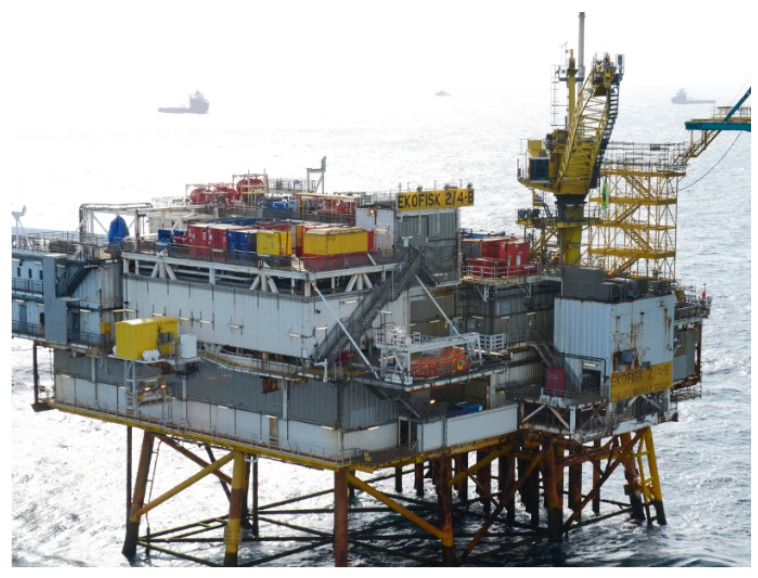
Ekofisk 2/4 B platform is the longest-standing fixed offshore platform in the world, which lies in the North Sea and operated by ConocoPhillips. (Permission to use image was obtained from Dennis Nuss of ConocoPhillips. Photo Credit: ConocoPhillips).

**Figure A1:**
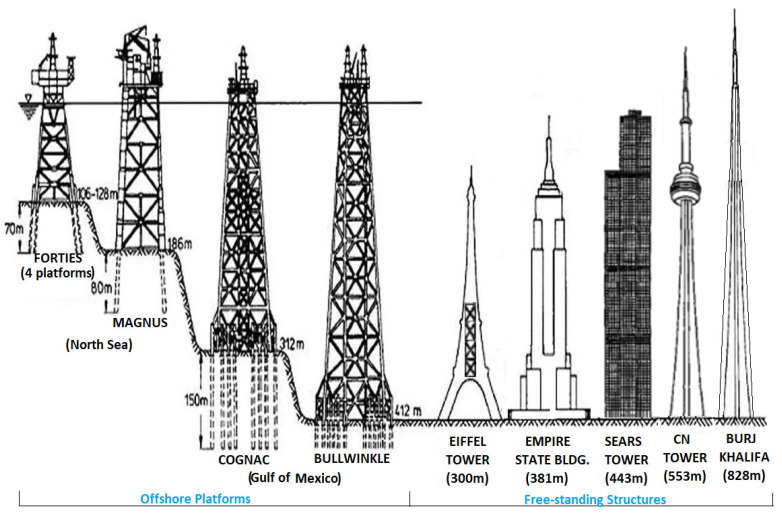
Typical structures for asset integrity management, and monitoring. It shows the comparative height of the tallest free-standing structures against the offshore platform [Credit: C.V.A.].

The maintenance management system should, in theory, be contained within an organization’s database with a full history of the operation, design, assessment, inspection, and maintenance records available to all essential staff. The maintenance of offshore structures is very important to ensure the structure has a long service life, most especially, aging structures. Extending the life of operation facilities beyond their design life poses safety, business, and operational hazards to the oil and gas sector. These risks have a substantial impact on business decisions and must be measured and controlled to ensure that these oil and gas facilities are still operational while they are aging. Hence, there are routine checks, audits, monitoring activities, maintenance regimes, and necessary conformity to industry standards that must be met [24,25,26,27,28]. Figure 9 shows ConocoPhillips’ Ekofisk 2/4 B platform, which is currently the longest-standing fixed offshore platform in the world and it is still well maintained and operational [7,87]. Table 2 shows a comprehensive application of sustainable maintenance management that is found on some longest-standing fixed offshore platforms. Some of these structures are illustrated in Figure A1 in Appendix A.

### 2.8. Asset Integrity Management

**Figure 10:**
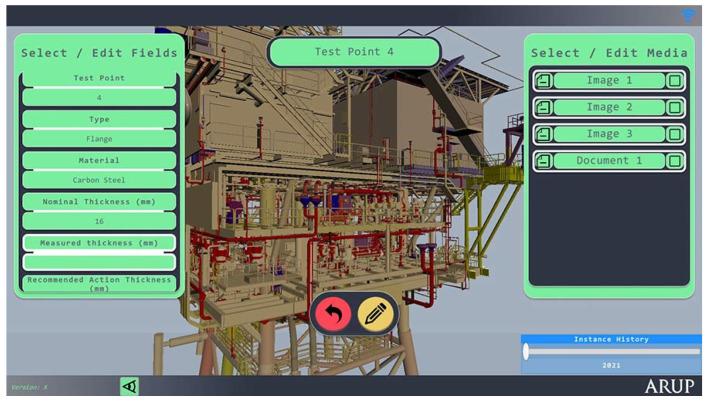
Typical integrity management application showing an offshore platform being inspected for the piping wall thickness using an in-house software by Arup. (Permission to use image was obtained from Cameron Dunn and Will Cavendish of Arup. Image Courtesy: Arup. Source: [127]).

Asset integrity, also known as asset integrity management systems (AIMS), refers to an asset’s ability to operate efficiently and accurately while also safeguarding the health and safety of all personnel and equipment with which it comes into contact, as well as the safeguards that are in place to ensure the asset’s long-term viability. Hence, asset integrity can be taken as the whole life cycle of an asset, from conception to decommissioning and replacement. Asset integrity management has evolved over the last few decades, from simple time-based inspections of key equipment to risk and reliability-centered management systems for all safety-critical parts. Any current asset integrity management policy must include risk-based inspection (RBI) and reliability-centered maintenance (RCM) technologies. However, many of these systems are qualitative, requiring each evaluation to be conducted by a multi-discipline expert team. These technologies have advantages and disadvantages, including the fact that they are time demanding and so slow to adapt to changes in process chemistry or operating procedures. Modern digital technologies provide a very reliable means of managing asset integrity in real-time. It is possible to determine the real-time status of essential portions of the asset by connecting information systems such as corrosion monitoring systems (i.e., online thickness measuring systems, corrosion probes, etc.) to the RBI and RCM systems, or digital control system (DCS) for the control room [45]. Thus, it is possible to determine the real-time status of essential portions of the asset by connecting information systems such as corrosion monitoring systems (i.e., corrosion probes, online thickness measuring systems, etc.) to the RBI and RCM systems, or digital control system (DCS) for the control room. When predictive RBI and RCM tools are linked to real-time operational data, it is possible to predict the impact of changing operating factors as they occur. Linking data from the DCS temperature and pressure indicators to an RBI creep life prediction algorithm, for example, would allow for the impact of real thermal history on the equipment’s projected creep life. Real-time asset integrity monitoring would open a lot of possibilities for flexible plant operations, including refining opportunity crudes. Using DCS pressure and temperature data, as well as crude assay data and online corrosion monitoring data (e.g., corrosion probe or field signature method data), crude feedstock blending could be used to allow for the refining of corrosive crude stocks without causing significant damage to plant infrastructure [45]. Although the application of appropriate techniques and new technologies is a crucial part of the overall integrity management process, there may not be a single right way or process to assure structural integrity, therefore, consensus may not always be obtained. However, the successful implementation and continuation of asset integrity management program is highly dependent on the operators’ understanding of risks and potential consequences, ensuring that integrity personnel that are engaged in integrity operations are involved in the same programs and that they are well comprehended by interested parties throughout organizations. The typical integrity management application on an offshore platform is presented in Figure 10.

While there are more AIM systems on the market than at any other time in history, no one-size-fits-all solution exists. Although no inspection plan or database can address all AIM concerns, integrity systems are still viewed as distinct from the rest of the operations [127,206]. Employees may be hesitant to take responsibility for their obligations, viewing the suite of AIM products as a company’s attempt to police them rather than an essential part of their job. However, the gaps in AIM packages must be filled with the vigilance of the same individuals they are supposed to protect, but a creative way may be required to get this message out. Hence, the system must be maintained by proper supervision.

### 2.9. Techno-Economic Report

Engineering drawings, design documentations, and equipment blueprints must be present and accessible for all assets. These documents make it easy to the project managers and design teams to properly plan the maintenance regimes of offshore platforms. They also permit them to successfully design at all phases of the asset life-cycle and in connection to the management of aging life extension. Every engineering activity that takes place during an asset’s expected service life should consider life extension concerns. One such concern could include the disruptive change in carbon emission by decarbonization. Hence, one concern of engineering designs is change, such as maritime application whereby shipping vessels and service offshore vessels (SOVs) are designed with the view to reduce carbon emissions by using more rechargeable batteries.

Techno-economic reports are a very important aspect of any offshore asset. It includes the cost for man-power, the processes for each operation, the cost of software, analysis cost implication, testing, validation, consultancy, inspections, auditing, and other levels of operation that will be undertaken. However, different scenarios should be considered in preparing the economic analysis, such as on offshore wind farms or crude oil prices. Based on the latter, the following scenario can be considered:

1. For three (3) alternative crude oil price options, there will be no further production enhancement action.
2. There will be the shortest extension period for various crude oil price choices.
3. For various crude oil price possibilities, the longest extension period is calculated based on the longest remnant life of a discipline.
4. There are three additional scenarios for extending the period between the shortest and longest periods for various crude oil price alternatives.
5. Capital expenditure (CAPEX) and operational expenditure (OPEX) sensitivity analysis for a variety of scenarios.

### 2.10. Safe Practices on Asset Management

In 2003, a Directive on Safety of Offshore Oil and Gas Operations was released by the European Union (2013/30/EU), which was an addition to other guidelines in the sector [208,209,210]. The EU admits that the current regulatory system is “divergent and fragmented” and that current procedures do not adequately guarantee that the danger of offshore incidents is reduced. The goal is to lessen the likelihood of major accidents that are connected to oil and gas production and to limit the effects of those catastrophes. The guideline has a stronger environmental component than just safety. It will apply to both current installations and activities for offshore oil and gas as well as future installations. Given that EU-headquartered operators will need to undertake operations within and outside of Member States’ offshore waters in compliance with their Major Accident Prevention Policy (MAPP), it offers far-reaching ramifications. Additionally, even if accidents occur outside of EU seas, enterprises that are registered in EU nations must report them if they occur on their installations. Additionally, it is necessary in European waters. Similar to the North Sea regime, the new rule will need independent verification and will apply to both safety-critical elements and environmentally-critical elements.

HSE guidelines for work safety recommend that sufficient precautions are taken by conducting risk assessments by considering all the potential risks, the hazard at workplace, and the safety regimes to consider [208,209,210]. It provides information for any corporation that is efficient, productive, and well-managed will also do well in terms of safety. The data that are obtained from asset management is only useful if it is of high quality and if it can be analyzed onshore. The engineering discipline is technical with skills shortages, which is a global issue that affects a wide range of sectors. Recent low oil prices in the 2016 and the global COVID19 pandemic in 2019–2021 forced the sector to lay off a large number of offshore and onshore personnel, resulting in a loss of skills and experience. Since maintenance resourcing and staffing were frequently targeted for cost-cutting, skilled individuals lost faith in the industry as a promising long-term career path. The situation has been compounded by a general shortage of skilled labor, owing to the decline of onshore heavy industries, which once offered ready sources of skilled labor.

**Figure 11:**
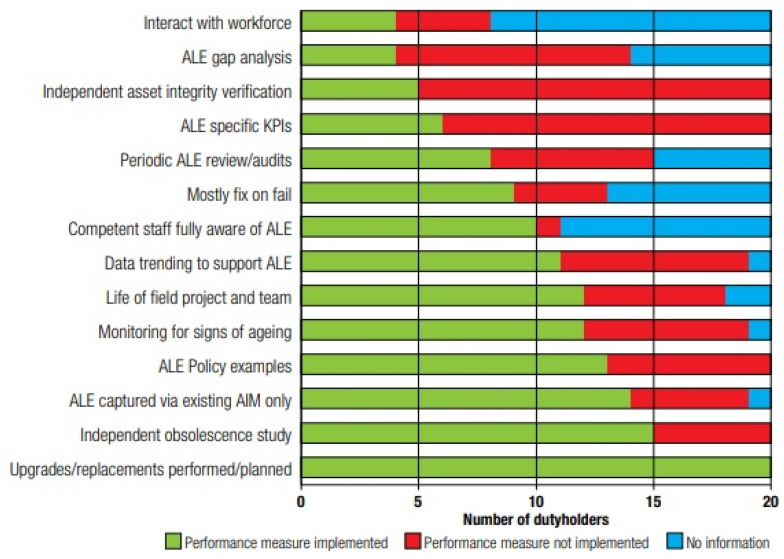
Typical inspection report performance factors showing good practice performance measures distilled from the electrical, control, and instrumentation inspections (this image is re-used/reproduced with permission of the Health and Safety Executive under the terms of the Open Government License, Courtesy: HSE, UK. Source: [189]).

During this time, some companies looked at the economic life expectancy and devised a strategy to sell assets. In many cases, this resulted in short-termism in maintenance planning, which decreased the plant’s overall condition, particularly the fabric. This short-term approach also over-looks the fact that some assets have a strategic purpose that could be useful to others in the future. This raises safety and sustainability concerns about whether there is sufficient investment in crucial installation maintenance to ensure long-term viability. Experiences from various offshore facilities are used to make policy recommendations and findings on the lessons of asset management [208,209,210,211,212]. Additional discussions are presented in related studies in this subject area [189,213,214,215,216,217,218,219,220,221,222]. Typical inspection report performance factors are presented in Figure 11.

## 3. Sustainable Elements of Asset Management

This section presents some sustainable asset management on the offshore platforms.

### 3.1. Human Factors

**Figure 12:**
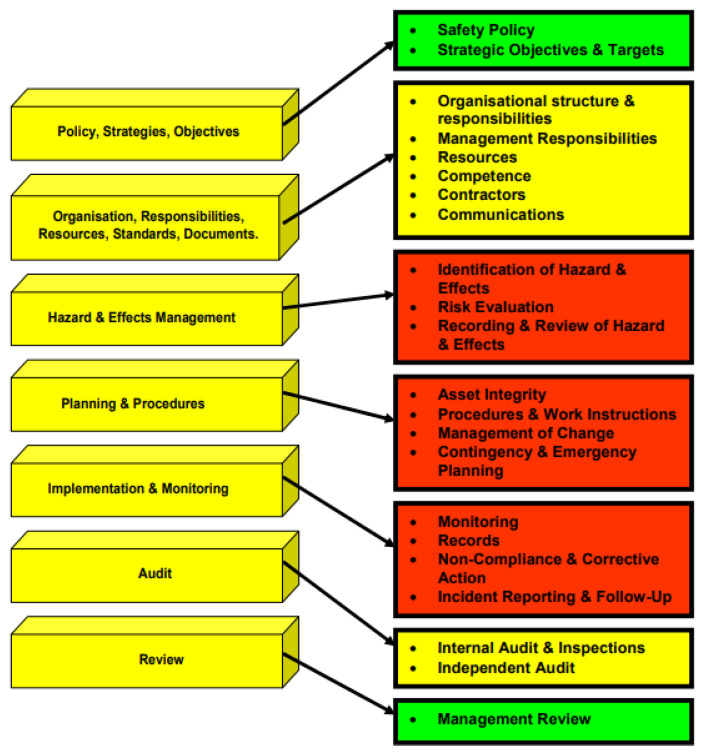
Elements of a management system that is capable of managing aging plant issues (This image is re-used/reproduced with permission of the Health and Safety Executive under the terms of the Open Government License, Courtesy: HSE, UK. Source: [224]).

Aging structures require certain elements of management [189,221,222,223,224]. To achieve effective and safe operations, the human factor domain includes methodologies and information that may be used to examine and enhance the interaction between people, technology, and organizations. When changes are made or when the established human (individual), technological (job), and organizational framework is challenged, a study on human factors should be conducted. However, daily operations on offshore facilities involve some risks thus the need for various guidance on offshore facilities by respective bodies [225,226,227,228,229,230,231,232,233,234,235,236]. Other procedures that could be conducted are included in the elements of a management system for aging facilities that was proposed by HSE in Figure 12.

**Figure 13:**
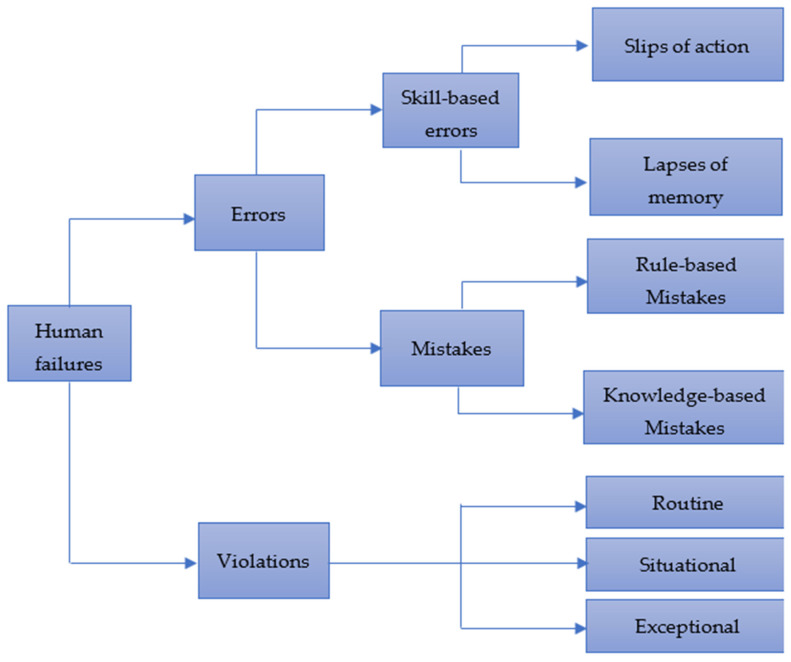
Different types of Human Failures.

Organizational structure, competency or training requirements, and succession planning should all be considered. The human element is an important factor in asset integrity. An understanding of human factors is seen in human errors, mistakes, and other types of human failures. Asset management uses human factors in identifying causes of accidents, prevention of accidents, and the design of effective control measures. Figure 13 illustrates the different types of human failure.

By definition, human factors are defined by the HSE as “environmental, organizational, and job elements, as well as human and individual qualities, which impact behavior at work in a way that can affect health and safety” [225]. This can be improved upon by considering three components when considering human factors: the work, the individual, and the organization, and how they affect people’s safety- and health-related behavior, as detailed in Section 3.2.

### 3.2. Organizational Factors

**Figure 14:**
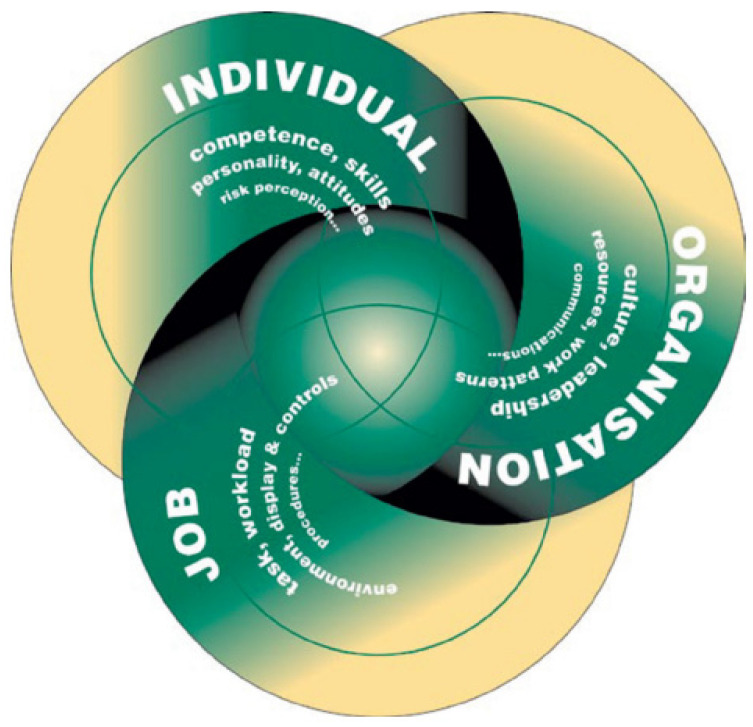
Some elements of human factors in occupational health and safety. (This image is re-used/reproduced with permission of the Health and Safety Executive under the terms of the Open Government License, Courtesy: HSE, UK. Source: [225]).

Organizational factors are important in the management of offshore structures. Engineering design, contract, and procurement management are all parts of an organization system that must be considered. Asset aging and life extension factors must be carefully considered in engineering design and related procurement operations. Before deciding on the adoption of steps, the risk that is posed by each result as well as the aggregate potential (future) dangers must be assessed. Figure 14 illustrates the relationship between the leadership in an organization.

The task—To account for human performance limitations and strengths should be created in accordance with ergonomic principles. Making sure that each employee has the right job for them will prevent overwork and guarantee that they make the best possible contribution to the company. Physical compatibility also takes into account the layout of the entire office and working space. The individual’s knowledge and decision-making needs, as well as how they perceive the tasks and hazards, are all factors in mental match. Human mistake is a possibility when job requirements are out of alignment with people’s talents.

The individual—Depending on the demands of the task, people bring their own attitudes, abilities, habits, and personalities to their jobs, which can either be strengths or disadvantages. Individual traits have a complicated and considerable impact on behavior. Their detrimental impacts on task performance might not always be offset by job design. Some traits, such as personality, are immutable and cannot be altered. Others, including abilities and attitudes, can be improved or modified.

The organization—Organizational factors have the largest impact on both individual and group behavior, but they are frequently disregarded in both work-related task design and accident and incident investigation. Organizations must create their own thriving cultures of health and safety. Employee engagement and dedication must be encouraged at all levels of the organization’s culture, with a clear message that deviance from accepted health and safety standards is unacceptable.

### 3.3. The Learning Organization

Most oil businesses want to operate safely, create revenue, work economically, and provide a safe working environment. To achieve this, occupational procedure must be in accordance with the relevant standards. One of the industry regulations to OGPs as given by HSE bodies such as OHSAS include recording/reporting every incident, occupational illnesses, all injuries, and mishaps that are avoided. This also includes the attitudes of the workers in order to improve operational resiliency and reliability; the progress of a learning organization begins with learning; the possibility of unexpected events can be reduced by being curious about how work is done, being aware of risks, and committing to predicting errors.

**Figure 15:**
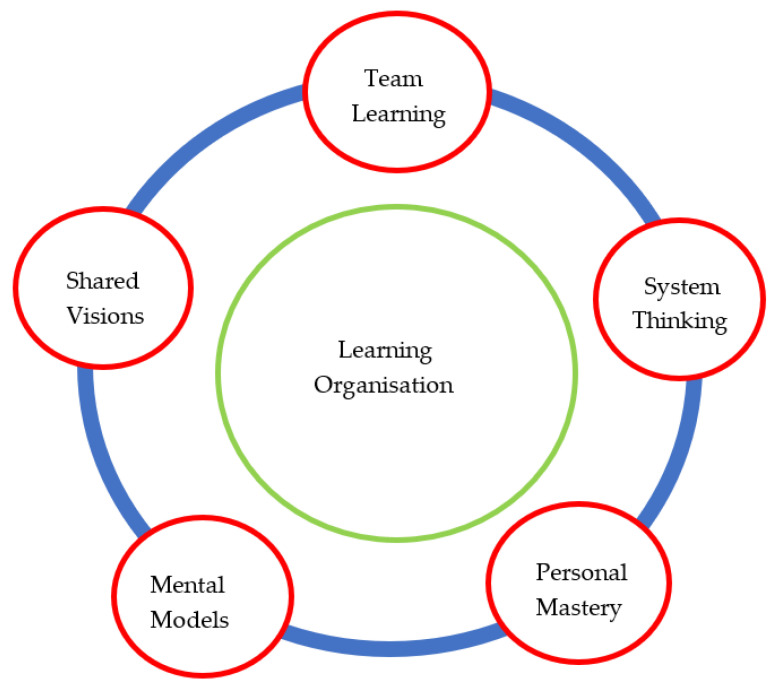
Five disciplines of a learning organization.

A learning organization is always looking for new methods to improve its safety, efficiency, and responsibility. A learning organization examines interactions among people, equipment, and work processes in order to reduce human error. A learning organization conducts rigorous investigations into all significant accidents in order to determine the root cause and share lessons that have been learned with others around the world in order to enhance our procedures, training, maintenance programs, and designs. A learning company can improve their ability to safely manage work and critical activities by using human performance principles and a learning mindset. This is represented in five disciplines of a learning organization, depicted in Figure 15.

**Figure 16:**
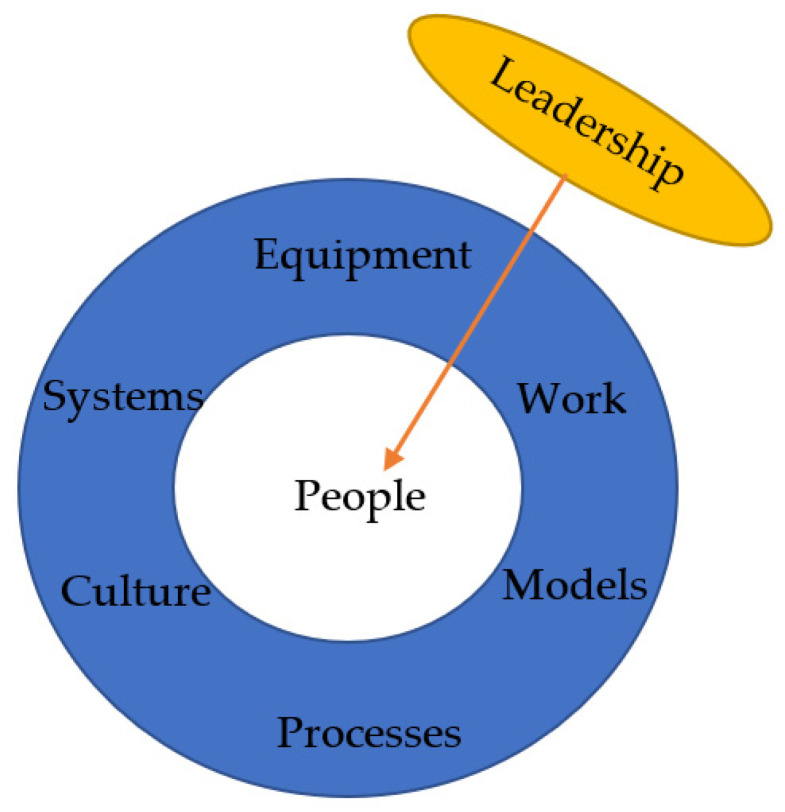
The relationship between the leadership in an organization and the people.

A learning organization has procedures in place to encourage open and honest discussion of the work at hand and the exchange of ideas. Learning teams are facilitated sessions in which the facilitator and team debate unexpected incidents or successful work events to gain a deeper understanding of the nuances in which the work was completed. Following an incident or near miss, the “Opportunity to Learn” procedure allows information to be immediately shared so that lessons learned can be identified and implemented towards relevant areas to prevent similar problems. Additional actions such as verification of personal and process safety precautions, as well as genuine leadership engagement with field operations, enhance this strategy. Figure 16 illustrates the relationship between the leadership in an organization and the people.

### 3.4. Personal and Occupational Health

**Figure 17:**
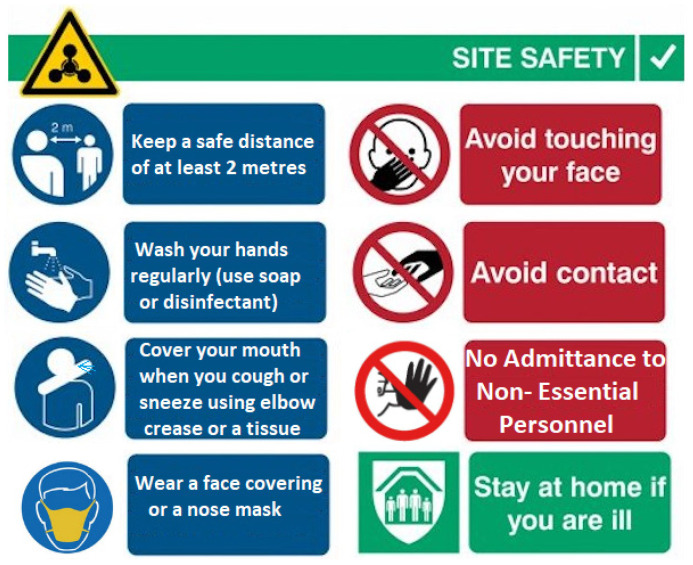
Typical COVID19 prevention signs for offshore facility site safety.

Prior to adopting ALE, the OGP will assess the current state of working environment elements that are relevant to life extension. There are some considerations on occupational health that are highly relevant to offshore platforms. The factors to consider are as follows: lodging facilities, outdoor operations, storage, material handling, noise/vibration pollution, ergonomics, ventilation, lighting, radiation exposure, chemical exposure, biological hazards, and epidemics or pandemics. Recently, there was high prevalence of Coronavirus globally during the COVID19 pandemic of 2019–2022 period, both pre-COVID19 [237,238,239,240,241,242,243,244,245,246,247] and post-COVID19 [248,249,250,251,252,253]. These studies show that the pandemic had a huge effect on the global economy and all sectors. Typical COVID19 prevention signs for offshore facility site safety are given in Figure 17.

The key goal of occupational health evaluation is to determine the current state of the working environment in terms of both operational and technical requirements. The assessment/evaluation is based on the current conditions at the facilities, and if necessary, further evaluations and assessments are conducted as needed. Before deciding on the adoption of actions to improve the working environment, the operational risks of every finding, as well as prospective dangers, must be assessed.

### 3.5. Health, Safety, and Environment (HSE) Management System

The Health, Safety, and Environment (HSE) Management System Standard ensures that corporate operations are handled in a safe, healthy, ecologically, and socially responsible manner around the world. The main industry standards, such as ISO 9001, ISO 14001, ISO 45001, and OHSAS 18001, are the current standards that all oil operators should abide by, align with, and operate upon. Furthermore, these companies have their own set of corporate standards and guidelines called Standard Operating Procedure (SOP). Each division should maintain the HSE Management System of the corporation in compliance with their corporate standard to identify and manage the local operational risks to the business, stakeholders, contractors, employees, and the planet. Taking good note of the ecosystem, the climate, and the entire environment should be considered a sustainable aspect of management.

**Figure 18:**
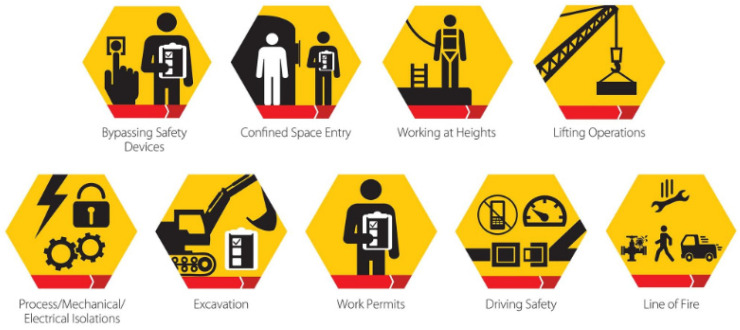
Typical safety signs that are used on offshore platforms for the oil and gas industry.

Each division is responsible for incorporating HSE and sustainability issues into decision-making activities, day-to-day operations, project planning, project development, and schedule planning by periodically reviewing their HSE management systems against the corporate standard. They assess the current situation, identify opportunities for improvement, and then put into place important activities to decrease risk and improve HSE performance. Having a yearly performance evaluation is used to assess their output, track project progress and check the accountability of each division. To ensure effective HSE performance, annual objectives, targets, and deadlines are defined and tracked, the leadership is kept up to date on the progress by having performance reports [24,25,26,27,28]. Figure 18 depicts some of the typical safety signs that are used on offshore platforms.

### 3.6. Process Safety

Process safety is achieved by employing extra measures, or barriers, and precautionary measures to keep offshore facilities safe, as well as oil and gas products safe and controlled, thereby reducing the risk of harm to people, property, or the environment. By definition, a process safety event is defined as an unintended or uncontrolled release of any substance from a process system. Process safety occurrences should be prevented, controlled, and mitigated by the oil firm using consistent practices and processes. Active, passive, or procedural barriers can be effectively used, and they can include equipment and/or people. Depending on the degree of the possible threat, the oil company should employ different barriers to establish redundancy and establish risk assessments [226,227,228,229,230,231,232,233,234,235,236].

Throughout the organization, we strive to improve our process safety culture and performance. Process safety specialists from around the world gather regularly to share information and debate best practices for continuous improvement. To improve the safety of work processes on offshore facilities, engineers use new information and technologies to create safer systems. Also, to prevent process dangers and preserve asset integrity, trained operations employees execute routine maintenance. Experts in process safety examine incidents and communicate their findings around the world. 

One of the main goals is to raise process safety knowledge and expertise within an organization. Guidelines should be elaborated for process safety (such as Process Safety Fundamental) [24,25,26,27,28]. These guidelines should be established to promote process safety awareness by being good, executable, basic, and clear operating procedures. People become numb to the risks that they face over time, making errors more likely. These Process Safety guidelines should be designed to raise focus on important tasks in the recognition of these risks on offshore facilities.

### 3.7. Operational Factors

**Figure 19:**
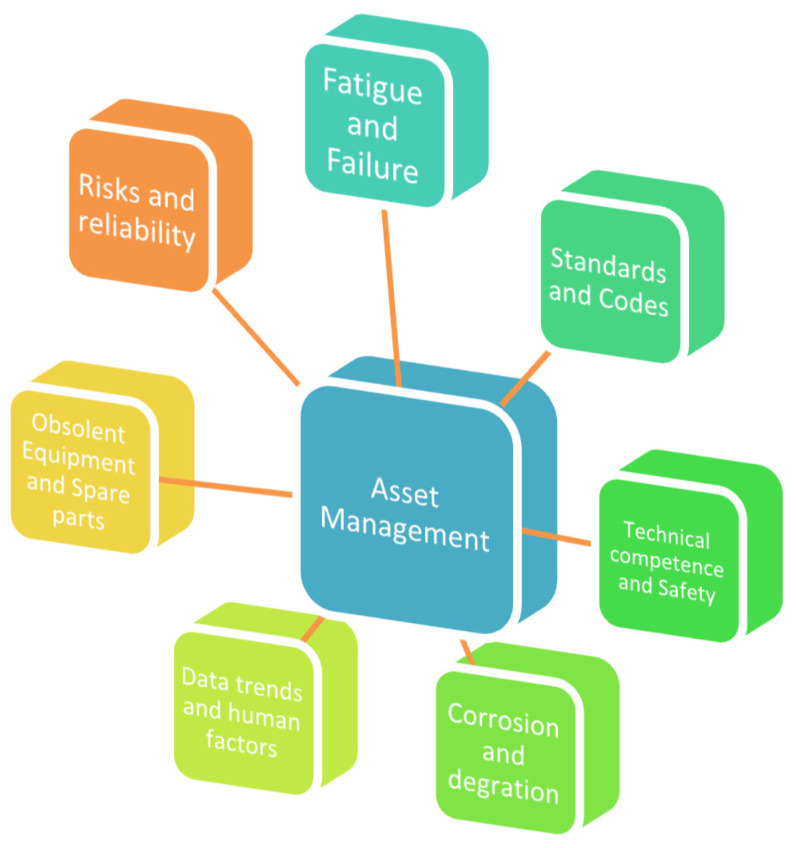
Different factors that are considered for maintenance management of offshore facilities.

Assets and equipment that are getting older pose more challenges in terms of maintaining equipment integrity, so they must be handled properly, as illustrated in Figure 19.

These could be the result of long-term deterioration and dangers, such as:

- Introducing new (or foreign) materials into production systems (such as marine riser fluids, pigging fluids, off-spec water injection, chemical tracers, downhole sand consolidation, and chemicals for enhanced oil recovery (EOR), etc.).
- Modifications to engineering standards and design codes.
- Degraded construction materials due to mechanisms that are related to corrosion.
- Mechanisms of cracks, fatigue, wear, or erosion.
- Mechanisms of ‘slow burning’ deterioration or degradation.
- Equipment obsolescence leading to a probable shortage of spares, excessive replacement costs, etc.
- Failure to record the accurate state of safety critical elements (SCE) throughout time.
- Inadequate data trends to estimate future hazards to safety and business continuity.
- Inadequate data trends for forecasting the probability of risks, reliability, and other failure assessments of the offshore structure or asset.
- Failure to normalize deviance that is related to human factors (accepting degraded conditions as the ‘new normal’).
- Lack of technical expertise in the industry, which is a combination of experience, training, qualifications, and competence.

### 3.8. Assurance and Verification

**Figure 20:**
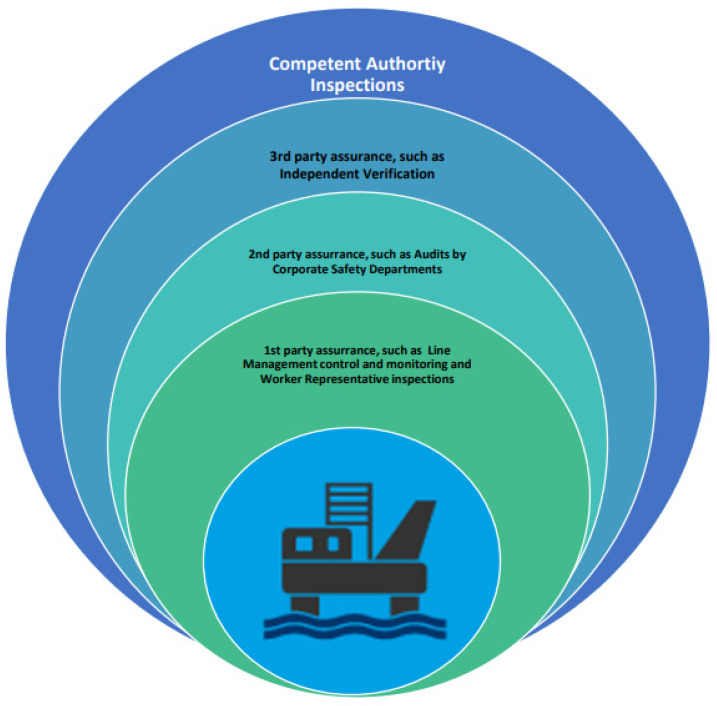
Assurance, monitoring, and assessment systems in the offshore oil & gas industry. (This image is re-used/reproduced with permission of the Health and Safety Executive under the terms of the Open Government License, Courtesy: HSE, UK).

**Figure A2:**
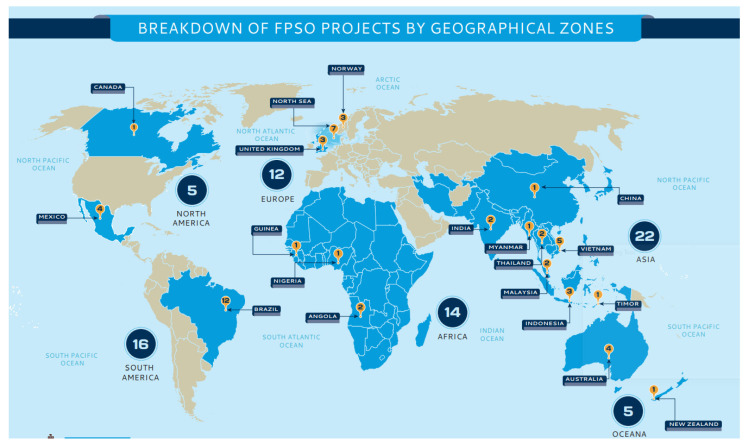
Map of breakdown of FPSO projects by geographical zones, showing various assets that are monitored; from FPSO Asia as of 2013.

The OGP is responsible for ensuring that previous experience with lifespan extension from other installations and operating locations is applied to the application’s analyses and evaluations. The application document must include any specific relevant information. OGPs are responsible for ensuring that analyses and evaluations are carried out in accordance with regulations, company standards, and have been verified by the necessary technical competent agency or authority. A model for assurance and competent authorities for monitoring, inspections, and verification are given in Figure 20. This process can also be conducted on floating structures, such as FPSOs, which can be monitored as seen in Figure A2.

### 3.9. Audits and Monitoring

HSE auditors conduct audits and inspections in various divisions of an offshore facility. They are in charge of managing and maintaining a process that provides objective, consistent, and independent assessments of the oil corporation’s operations; its conformity to key policies; and adherence to HSE rules and regulations. Further auditing methods exist inside business units to assess compliance with appropriate corporate HSE and regulatory obligations. The result of corrective actions from audits, changes that are made to their operational procedures, and other risk improvement items should be reported annually. This report could be made through a procedure that is designed to ensure that items are communicated to all levels of corporate management and are resolved quickly. Based on appropriate suggestions from regular meetings, the corrective actions from audits can be achieved. Lastly, these audits can be used to develop the process and report on risk management for obsolescence risks (OR), or sustainable development (SD). Table 3 presents a typical report that is used to define obsolescence risks with recommended actions.

### 3.10. Asset Integrity Management on Pipelines

**Figure 21:**
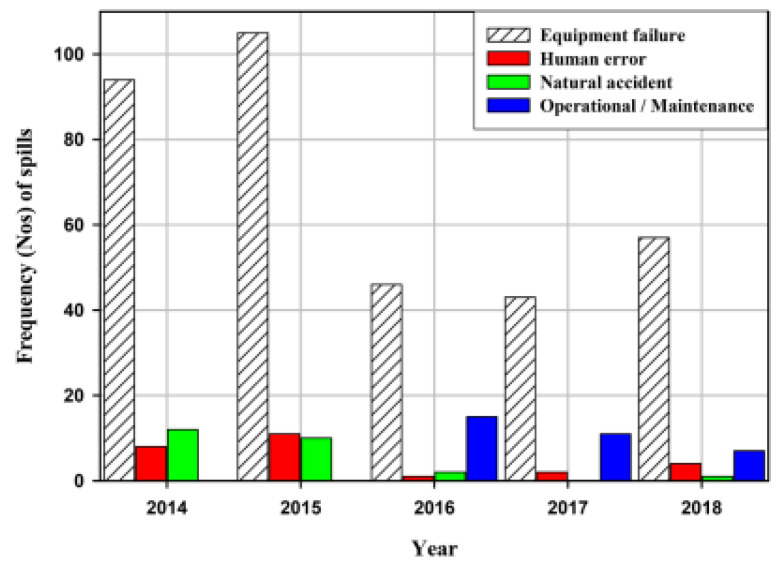
Different factors that were considered for failures of offshore pipelines due to equipment failure, human factor, natural accident, and operation/maintenance. (Image was reused with permission of authors – Prof. Chinwuba Victor Ossia and Engr. Augustine E. Agomuoh; and used under WJET open access rules. Publishers: SCIRP Publishers, Copyright year: 2021, source: [253]).

**Figure 22:**
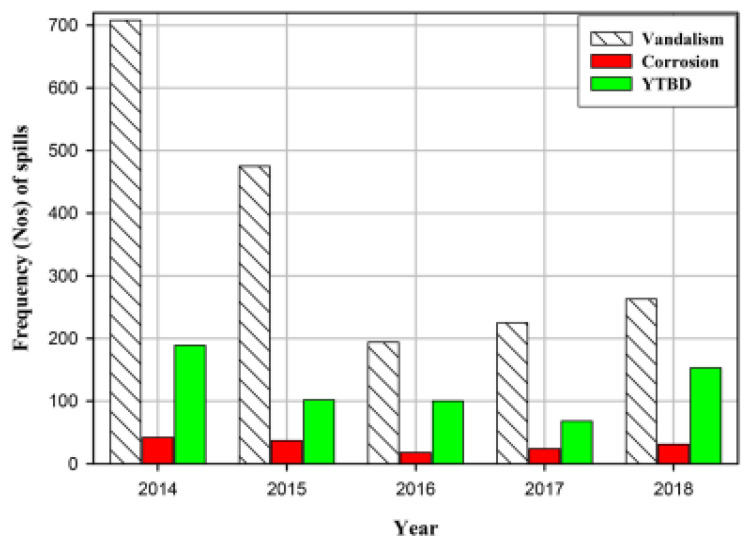
Different factors that were considered for failures of offshore pipelines due to vandalism, corrosion and yet-to-be-determined (YTBD) factors. (Image was reused with permission of authors – Prof. Chinwuba Victor Ossia and Engr. Augustine E. Agomuoh; and used under WJET open access rules. Publishers: SCIRP Publishers, Copyright year: 2021, source: [253]).

Oil and gas corporations are usually managed using a structure, which ensures that it operates over long time, and this could strain the design capacity. Agomuoh et al. [253] conducted an investigation on asset integrity management in the Niger Delta region, by looking at deep burial solutions to mitigate oil and gas pipeline vandalism. The study was able to ascertain that some faults are threating the oil and gas assets in these regions, particularly based on equipment failure, human error, natural accidents, operational/maintenance issues, vandalism, corrosion, and some yet-to-be-determined (YTBD), as seen in Figure 21 and Figure 22.

According to IQ [254], asset integrity is based on the assumption that the majority of employees in the firm will do the right thing, and no matter how hopeful that seems, most maintenance, inspection, and data management is done with the best of intentions. However, things are frequently not completed completely or in a timely manner. Simple remedies such as more regular inspections will not guarantee to catch every missed issue, nor will they inspire excitement if personnel are forced to increase their inspection work or are implicated in missed defects. Vandalism incidences began to rise steadily but gradually after 2016, and this trend has continued to this day [253]. The fact that corrosion-related failures remained flat (constant) as expected in Figure 1 is a reasonable testament to the efficiency of the integrity management systems in the IOCs that are assessed.

A significant portion of failures, as seen in Figure 21, were linked to “yet-to-be-determined (YTBD)” variables. The studies also revealed that for 2014, 2015, 2016, 2017, and 2018 consecutively, the spills that were attributable to yet-to-be-determined (YTBD) variables were 17.3%, 13.5%, 23.0%, 15.85%, and 26.88%, respectively. Experience suggests that these YTBD concerns are contested vandalism problems. Probably none of the spill inspectors could agree to call them vandals. Therefore, a paradigm shift in policy is required to recognize pipeline vandalism as a significant issue with pipeline integrity in Nigeria. The impact of additional pipeline failure causes (equipment, human error, natural disasters, and operations/maintenance failures) is illustrated in Figure 22. In compared to failure that is caused by natural accidents, and failure due to causes that could be characterized as internal to the pipeline operating firms is higher. This implies that the pipeline operating businesses must maintain the careful application of their pipeline integrity management systems and continue to make investments in new technology and instruments.

## 4. Proposed Guidelines and Policy Implications

**Figure 23:**
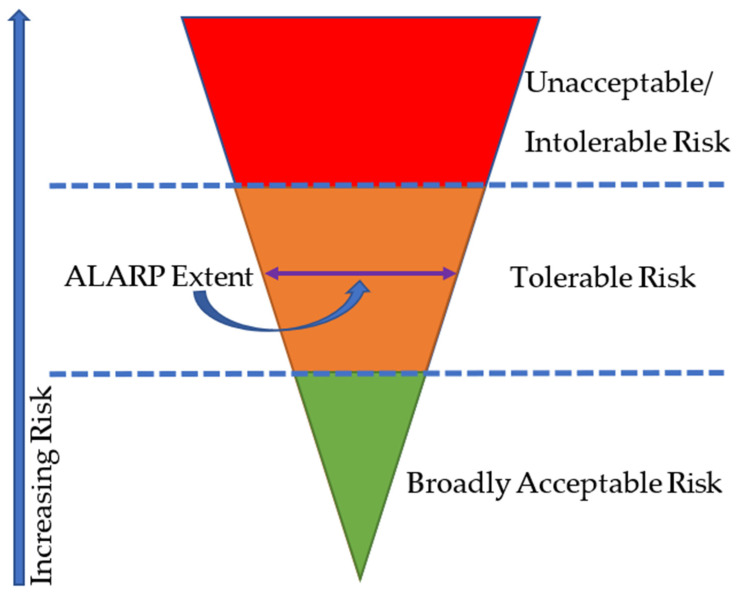
The ALARP Principle showing levels of tolerance to risk.

The section presents the proposed guidelines from lessons that were learned and policy implications for managing assets in oil and gas platforms. Each of these assets are usually managed using an asset management system to ensure that the offshore structure or onshore structure operates within the design capacity. These offshore structures are expected to be safe under the period of its service life or extended life. When it comes to offering an asset life extension solution for aged offshore or onshore assets, there are numerous elements to consider. Much of the infrastructure in the oil and gas sector is currently approaching or is past its operational life expectancy. Most of the of the offshore assets have a service life of about 25 years, so oil and gas producers are frequently compelled to operate over their design capacity but are expected to do so safely. When it comes to offering an asset life extension solution for aged offshore or onshore assets, there are numerous elements to consider. This study outlines the major considerations and the steps to take when evaluating asset life extensions for an aging offshore structure (or asset). These assets must meet the ALARP (As Low As Reasonably Practical) requirements as a minimum for each field and exhibit fitness for purpose at all stages of the asset life extension. Thus, the need to have asset assessments and asset integrity management [253]. The proposed ALARP tolerance showing typical tolerance limits is given in Figure 23.

The application of the ALARP principle could be seen as fulfilling the need to keep the risk level “as low as possible” if the ALARP evaluations are well-documented. In the ALARP zone (between “lower tolerable limit or broadly acceptable risk” and “upper tolerable limit or unacceptable/intolerable risk”), the risk is only acceptable if risk mitigation is either impractical or would cost much more than the benefit received. Utilizing cost-benefit analyses as the foundation for the choice of whether to execute specific risk-reduction measures is a typical method of determining what is realistic. If a risk exceeds the “upper bearable limit”, it may not be justified under any normal circumstances. In most cases, the “upper tolerated limit” is specified, while the “lower tolerable limit” may occasionally go unspecified. Assuming that ALARP evaluations of risk-reduction methods will always be necessary, this won’t prevent the methodology from being used effectively. Risks involving people, the environment, and assets can all be accepted under the ALARP principle.

Recognized standards and regulations must be adhered to whether risk reduction involves design, equipment selection, or operational measures, if they exist. Only when the risk is smaller than that arising from applying the standards or regulations may deviations occur. The elaboration of standards in this area is an important aspect of the developments achieved. Standards bodies like the International Standardisation Organisation (ISO) spend a lot of time drafting, formulating and publishing standards and guidelines. Technical advancements may, in some situations, make it difficult to apply standards and guidelines to the most up-to-date tools and working techniques, or they may prevent the use of solutions that minimize health and safety hazards in line with the ALARP principle. Therefore, when selecting a specific standard, it should always be taken into account if further risk reduction in accordance with the ALARP principle is feasible. While more than one standard contains requirements for the same health or safety requirement, the ALARP principle must be taken into consideration when selecting the standard.

However, research on asset management in the industry shows that oil and gas operators are participating in price-responsive strategies and asset optimization, according to study performed by Oil & Gas (IQ) [254]. In order to make ends meet, these businesses are increasingly re-evaluating their policies, particularly in the Gulf of Mexico (GoM), Offshore West Africa (OWA), and the North Sea (NS). According to IQ [254], more than half of oil and gas professionals currently work on installations that have been in service for more than 20 years, with less than a third working on facilities that have been in service for less than a decade. The report further stated that more than half of asset integrity professionals’ budgets have been reduced, and the average AIM rating of those professionals’ own companies is 5.4 out of 10. Only 52% said their workload was manageable in terms of fulfilling deadlines and maintaining safety, and the majority had a meagre budget of less than USD296,612.50 (as at 22 August 2022). The two most pressing challenges, according to asset integrity experts, are maintaining assets within budget and the age of the assets themselves. A lack of communication between departments is by far the most significant fault in oil and gas organizations, followed by a lack of safety culture.

However, a Safety Case can be undertaken on offshore facilities. By definition, a Safety Case can be defined as the document that describes the management system for safe operation of an offshore installation. It should demonstrate that all hazards have been identified and assessed and are under control by effective safety measures so that the exposure of personnel to the hazards has been minimized. However, there is the need for organizations to include key elements of asset management, and related ideals, such as health and safety [254,255,256,257], risk assessments [258,259,260,261,262,263,264,265,266], gap assessments [267,268,269,270,271], audit [272,273,274], life extension [275,276,277,278,279,280,281,282,283,284,285], asset integrity [39,286,287,288,289,290,291,292,293,294], Safety Critical Elements (SCE) [295,296,297,298,299,300], safety case [301,302,303,304,305,306,307,308,309,310,311], safe practice [312,313,314,315,316,317,318,319], asset management regulations [320,321,322,323,324,325,326], and general development of offshore assets [327,328,329,330,331,332].

In another report by HSE UK [221], it was recorded that leadership plays a critical role in improving comprehension, simplification, challenge, and learning, as well as performance during significant hazard controls. It was also reported that several concerns were identified that may be solved with better senior leadership. Management can get a comprehensive picture of the state of the plant and equipment is hampered by the complexity of many maintenance systems and the poor quality of maintenance data. The results of hardware and system testing were found to be a good indicator of the overall effectiveness of the maintenance systems. It can be used to help with plant efficiency and maintenance planning to improve productivity.

Learning can be accomplished through finding and sharing best practices, as well as having a mechanism in place to ensure that the learning is incorporated. Companies’ audit and review processes give means for identifying and sharing positive and negative performance. According to recent research, company audit arrangements are not being used effectively to learn about performance and share these learnings in many circumstances. Companies cannot address poor performance or discuss good performance without the intelligence to understand how they are performing. The auditing industry should think about how it can be used more efficiently. Improved learning is rarely effective if it is led by an independent installation; otherwise, it must be driven by the company. Companies must supply the impetus and the procedure to enable learning to be ingrained. Trade associations play a significant role in fostering learning in the industry.

The offshore industry has highly adapted to technology and the digital age. This has also led to the reduction of skilled personnel within the industry because some processes are automated using robotic arms, touch-screen automations, digital documentation and other programable machines. Hence, this has resulted in the dearth of plenty of technical work groups that made significant contributions to the early learning on significant hazard control over the past few decades. The Health and Safety Documentation for the implementation of monitoring, sustainable maintenance and safety practises must provide a choice of standards and guidelines. Where applicable, the operator or owner should apply harmonised standards when selecting an industry regulation or standard. This will assure adherence to the rules implementing related directives, and documentation of compliance can make reference to these rules from relevant standards bodies for harmonised standards that already exist. Lessons that are learned could be applied in fault diagnosis and monitoring systems for different onshore/offshore assets in the oil and gas industry towards developing proposed guidelines and policy documents in future research.

## 5. Sustainable Maintenance and Reliability-Centered Maintenance

**Figure 24:**
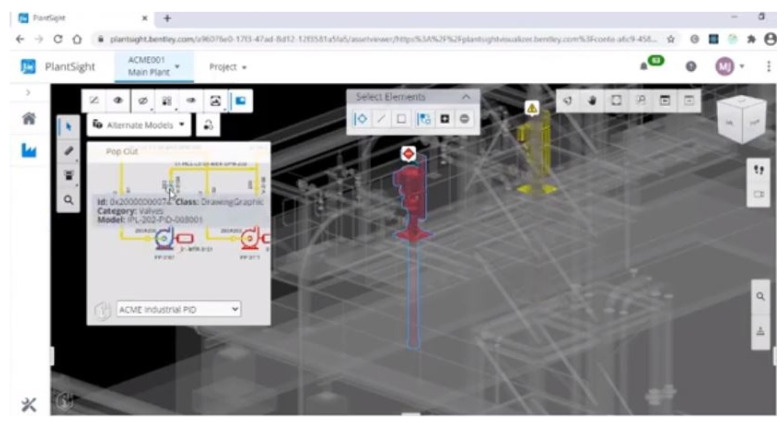
Typical asset integrity management using PlantSight by Bentley Systems.

The section presents an overview on managing assets in oil and gas platforms, and sustainable maintenance and reliability-centered maintenance. There are maintenance software packages for plants on offshore platforms which use reliability-centered maintenance (RCM), which is a strict and organised method, to maximise asset maintenance strategy. The procedure is based on the tried-and-tested analysis techniques that which are useful for extensively analysing the impacts of systemic failures. They include failure modes and effects analysis (FMEA) and failure modes effects and criticality analysis (FMECA) methodologies. The right maintenance actions to address each of the observed failure modes and their effects can be determined using RCM after system problems have been recognised. The capacity of RCM to account for the operational context in which the system is operating is one of its main advantages. This is one of the key factors affecting the system’s dependability. The system accounts for the whole plant integrity management using maintenance software solution. Application tools for asset management ensure sustainable maintenance and reliability is PlantSight by Bentley Systems which incorporates Siemens’ Digital iTwins for cloud performance, as in Figure 24. 

Plant integrity management makes ensuring you have the operational procedures, frameworks, instruments, skills, and resources required to maintain integrity across the course of an asset’s lifecycle. To properly manage costs and risks, design, operational, and technical integrity must all be carefully monitored. Stricter rules and increased safety knowledge are required for integrity management as the focus on process plant safety grows. Operators seek solutions that are both fully compliant and practical, able to meet their needs on a daily basis and solve real-world problems. DNV’s Asset Integrity Management (AIM) solution called Synergi Plant, provides a comprehensive plan-do-check-act methodology for managing risk either quantitatively or subjectively. These packages are designed with different industry standards for service, operation, reliability and management systems. For plant integrity management, DNV’s Synergi Plant software adheres to industry norms and best practices including ISO 55000, ISO 14224, DNV-RP-G101, API 581, IEC 61508, IEC 61511, ASME and API engineering formula. It should be noted that ISO 55001 is a framework for an asset management system rather than for asset management alone which is similar to ISO 9001 and ISO 1401, as detailed with other standards presented in Section 9. The base package offers the scalability to add additional software modules, such as reliability-centered maintenance (RCM), performance forecasts (RAM analysis), bespoke RBI, and safety integrity level (SIL), that correspond with the requirements of the client. These modules improve asset availability and dependability while reducing risks related to containment loss due to deterioration. They also protect the integrity of the assets. AIM plant maintenance standard packages are developed to assist owners and operators in beginning their road toward asset integrity. With the help of a risk-based analysis tool and a standard architecture and framework, these software packages offer comprehensive inspection data management system capability. They are more straightforward options with reduced ownership costs that address fundamental integrity requirements. With the application of these software, there are scalable solution to meet the asset integrity requirements of your business.

## 6. Dynamic Positioning Using Sensors on Offshore Facilities

On offshore facilities such as in Figure 1, there are different fault diagnostic components that are used and other monitoring applications, as detailed in this section. The application of sensors on offshore facilities includes monitoring as reflected in various studies on monitoring sensors, dynamic positioning sensors, condition monitoring sensors, and fault detection systems [121]. Firstly, studies on dynamic hypothesis testing for fault detection on offshore mooring lines was recently conducted by various researchers [333,334]. Based on GPS and motion sensors, Siréta and Zhang [334] applied an artificial neural network to identify mooring line defects on offshore units. In another study, floating offshore wind turbine (FOWT)’s mooring line fault detection was demonstrated utilizing a wave-excited linear model that was based on the Kalman filter algorithm for the JONSWAP spectrum [335]. Changes in parameters that cannot be directly monitored can be detected using Kalman filter techniques [336]. However, there are several uses for the Kalman filter techniques as seen in various studies which reflect the uses of the Kalman filter for defect detection [337,338,339]. Auger et al. [340] provided an overview of the Kalman filter’s industrial applications. In another study, some methods for condition monitoring of mooring lines that were used for offshore structures were presented [341]. Based on the application of the sensors, Imai et al. [342] illustrated offshore applications of the extended Kalman filter for structural dynamic systems, although hydrodynamic coefficient matrices with non-linear drag and linear inertia forces are identified for an offshore tower that is aroused by wave forces.

Dynamic positioning is a significantly broad area of application for the Kalman filter technique in marine applications. Motion control systems are used by the majority of contemporary maritime boats to hold a position or go along a desired course. The application of the Kalman filter for ship motion and course keeping control systems, position and heading regulation, route following, and trajectory tracking are all areas of research in this field. Zhao and Su [343] used an extended Kalman filter to estimate the moving horizon for a maritime dynamic positioning system. Perez [344] displayed a position and heading control system for ship course-keeping autopilots that applies wave filtering using Kalman filters. A VTOL aircraft landing is depicted in Triantafyllou et al. [345] that uses a Kalman filter to estimate the motion in real time for heave, pitch, roll, sway, and yaw. Other studies were identified that provide further Kalman filter applications for dynamic positioning [346,347,348]. Tockner et al. [349] published a feasibility study in order to demonstrate the viability of using the extended Kalman filter technique to detect flaws in dynamic systems with a large number of degrees of freedom.

Aside from these issues, there is the need for other monitoring methods for fault diagnosis on oil and gas assets. Significant forces may be generated on the platforms and in the connection elements by environmental conditions such as wind and waves [118,119,120,121,122,123,124,350]. The potential places where it is safe to erect such a platform structure are largely limited by these forces [125,126,127,128,129]. Designing the platform system for unshielded offshore areas is important to maintain a large number of potential locations [169,170,171]. This calls for extremely high force resistance in the connection elements as well as consideration of the motion hydrodynamics [157,350]. It is advantageous to identify potential problems in the platform connection parts as soon as feasible in order to ensure the dependability of the platform arrangement and to save servicing expenses. As there are only three ropes and at least twelve fenders per side of a platform, the pretensioned ropes that are made of synthetic fibers, are presumed to be the crucial components of the system in this study. Some benefits of synthetic fiber ropes include their lower weight, which makes them simpler to install, and they are grease-free [351]. Aside from these specific benefits, rope replacement decisions are made based on visual inspections and the number of load cycles [352]. Often, the only way to do a visual assessment is to demount the ropes. For investigators, offshore rope surveying can be dangerous and labor-intensive. In the platform configuration, where ropes pass via pipes inside the modules, dismantling the ropes is risky and takes a lot of work when done offshore.

## 7. Fault Monitoring Using Sensors on Offshore Facilities

Fault monitoring applications including monitoring for compression fatigue, heating, tensile fatigue, vibration/shock, abrasion, creep, UV radiation, and strain are the primary damage processes for fiber ropes [352]. Rope behaviors such as breaking strain, breaking stress, and stiffness alter as a result of rope fiber degradation and fatigue [341]. Unwanted dynamic behavior of the platform arrangement is caused by the fraying of the platform connecting ropes [341,349]. The platforms may no longer be suitable for the intended application due to increased strain on defective ropes that causes shocks and greater platform movement amplitudes. Continuous rope condition monitoring is extremely important to prevent this consequence. Gordelier et al. [353] demonstrated a variety of condition monitoring approaches for fiber ropes that were used in anchoring offshore applications, such as vibrational techniques, magnetic resonance, conductive internal elements, and fiber optics. Sensors inside the rope are important for some monitoring systems, necessitating a particular rope design that must not compromise the rope’s structural integrity. Finding actual rope values and establishing rope defects is another method. It is preferable to use indirect rope condition monitoring approaches because the exact platform design is still in the conceptual stage. As a result, Tockner et al. [349] investigated the key parameter for condition monitoring, which was selected as the estimated rope stiffness.

Non-linear parameter identification methods are taken into consideration because the platform configuration exhibits a substantially non-linear behavior. Over the past few decades, a large number of non-linear state observers have been created; some of their applications are shown in various studies [354,355,356,357]. The extended Kalman filter, which is based on a normal Kalman filter observer that is used for linearized systems, is one observer that may estimate states and parameters of a non-linear dynamic system using indirect data. By adjusting the noise parameters in real-time, Mu et al. [358] presented a solution to the extended Kalman filter’s instability issues. A substructure approach for the extended Kalman filter is presented by Koh et al. [359] to estimate the stiffness and damping coefficients of a structure. In the context of uncertainty, the Kalman filter offers estimates of unknown states and parameters. The platform arrangement’s known non-linear multi-body system, which is subject to some added uncertainties, is fed data by time-varying stochastic wave heights, and measurements are noisy. In light of these facts, the extended Kalman filter observer is used for the rope stiffness estimation in order to identify problematic ropes using acceleration data. The data are obtained at various places along parts of modular offshore platforms and modular multi-level converters [349,360,361,362]. This technique allows for the detection of faults in the rope connection elements, such as cracks or ruptured strands, as a change in the stiffness of the connection element.

## 8. Patents on Sensors for Monitoring Offshore Facilities

There are more developments on offshore monitoring systems, seen in inventions and publications in various areas that were earlier discussed in Section 6 and Section 7. However, another aspect of sensor application is presented in recent reviews on condition monitoring and fault diagnosis (CMFD) on offshore structures which include state-of-the-art applications and limitations of CMFD [121,363]. Some other reviews have been presented on structural health monitoring (SHM) systems for offshore platforms [171,364,365,366,367]. Different field monitoring projects can be seen in offshore platforms [367,368]. Another aspect is the mooring integrity management that has been reviewed by Gordon et al. [369]. However, depending on the scale of the project, sensors are deployed.

Some application of strain gauges on composite risers and pipelines have been achieved using stain gauges and other similar sensors that are called Fiber Bragg grating (FBG) sensors [370,371,372,373] and fiber optic sensors [374,375,376,377,378,379,380]. ENI E&P developed a sensor system that was based on fiber-optic technology to assess a riser’s performance and fatigue life [381]. The system is made up of the necessary surface equipment, a connectivity system, and an underwater sensor network. A riser fatigue monitoring approach was described by different studies [382,383,384], such as its application on a TTR of the deep-water GoM Spar that was described by Thethi et al. [384]. Fiber-optic sensors, which can be pre-installed on new risers or after-installed on existing risers, are used in the invention by Morrison and Dean [385] to measure the stress in steel catenary risers (SCRs). Marine risers can be installed with a standard SCR instrumentation, which includes strain gauges, motion sensors, and measurements of the flex joint angle. In addition to the riser top tension measurement and riser fatigue performance measurement, risers are vulnerable to the current in deep water since they are narrow structures. In order to properly monitor risers in the field, the response behavior and performance, such as the vortex-induced vibration (VIV) must be measured.

Allen and Pinto [386] created a different riser monitoring assembly to monitor and control a riser connecting subsea well machinery and a floating platform. Due to their outstanding performance and easier installation and operation than their rigid counterparts, flexible risers—composite constructions that are made of numerous metal and polymeric layers—are employed extensively in offshore platforms. Andersen et al. [387] showed that it is possible to measure strain in flexible risers using FBG sensor technology. This represents an application of monitoring systems for pipelines as well as other related components like mooring lines [388,389,390,391].

**Figure 25:**
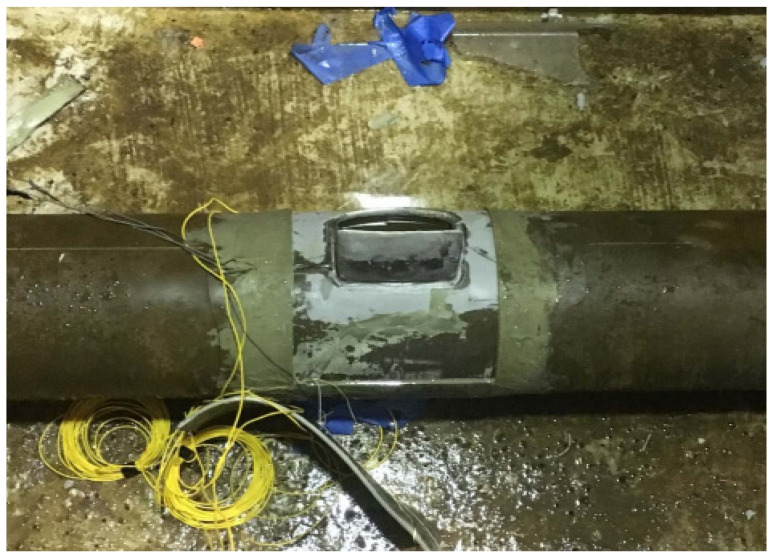
Typical composite pipe that is repaired with ComposiSleeveTM sample after pressure testing showing the attached optical fiber sensors (Reprinted/adapted with permission of Dr Chris Alexander. Copyright 2018 ASME & Chris Alexander. Source: Ref. [377]).

**Figure A3:**
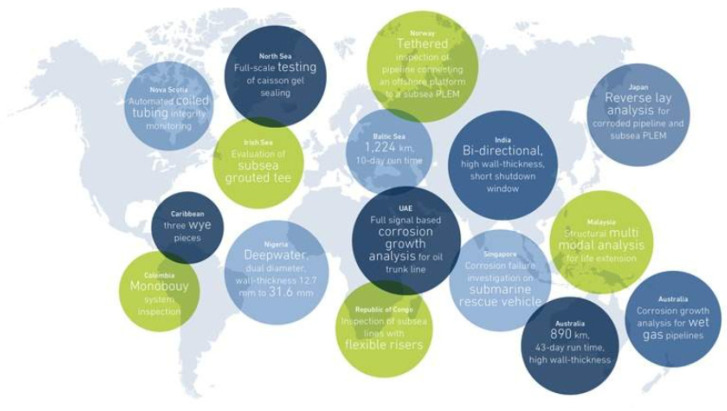
Map showing breakdown of projects for various assets that are located across geographical zones by an oil firm on Offshore Technology.

Using three prototype models with a bore diameter of 495.3 mm, Alexander et al. [387] investigated the performance of a composite-reinforced steel drilling riser for HPHT operating conditions. It was put through cyclic testing with a service temperature range of 180 °F to 32 °F, a 20-year service life, an internal pressure of 66,667 KN, a top tension capacity of 13,333 KN, and operating at 3048 m water depth. In another full-scale dynamic loading test, Jacques et al. [388] used non-destructive testing (NDT) methods on a flexible riser. Comparisons were made using the data that were collected by acoustic emission and Bragg grating-based fiber-optic sensors. Due to their high multiplexing capability, immunity to electromagnetic interference, little signal loss, small size, and resistance to corrosion, fiber-optic sensors have been widely used in field monitoring for offshore structures, according to studies [389,390,391]. These are applied for the integrity management for offshore assets. Typical examples are seen on various offshore structures, such as marine risers by oil firms such as the Rosen Group. This is identified in the map in Figure A3, showing the breakdown of projects for various assets that are monitored by geographical zones for an oil firm on Offshore Technology [392]. The map is used to show a range of offshore operations that could be conducted in maintaining different offshore assets. Also, some inventions identified for the application on offshore facilities, is represented in this list of some monitoring patents in Table 4. A typical application of a monitoring system using fiber optic sensors is utilised for testing the pipeline in Figure 25.

## 9. Standards on Asset Management and Sensors for Monitoring Offshore Facilities

It is important that asset management is conducted in accordance with the industry standards and specifications. Based on this study presenting a broad range of themes, there are multiple standards that include harmonized international standards that are appropriate for these applications. Also, each operation requires software application based on industry standards, hence the operator must select a particular set of recognised standards. Table 5 gives a list of some standards bodies while Table 6 lists some standards that are related to asset management, integrity, reliability and monitoring.

## 10. Conclusions

Currently, principles of asset management have been used in business modelling since asset integrity is now rivalling terms such as Agile, OPEX, CAPEX, and ROI as the buzzword on people’s radar. Secondly, with the cost of replacing assets and the resulting turnaround time being prohibitively high for so many facilities, there is the need to have guidelines on asset management. Thirdly, oil and gas companies are increasingly being pushed to operate beyond their initial design life and field life. Beyond these limits, asset life extension (ALE) poses new safety and business risk problems for the oil and gas industry. Operators face increasing problems in maintaining equipment reliability and integrity, as well as operating safety, as their equipment and facilities age. Hardware, as well as human and organizational variables, play a role in aging. Some factors to consider include corrosion, fatigue, erosion, obsolescence, normalization of deviance (accepting degraded circumstances as normal), changes to industry standards, and limited data to risk analysis and forecast future risks are all factors to consider. In cases where there is a huge fleet or many aging assets to manage, the difficulty is amplified.

General asset management of offshore facilities, pipeline inspection, assessment, and repair techniques are all aspects of offshore operations that require conformity to relevant standards. This paper presented some references, while it delves into integrity management strategies including codes and standards after a transitional overview on asset management. Subsequent sections discuss asset life extension models for risk-based inspection and suggested proactive actions. This study provides oil and gas managers with a guide to extending asset life, minimizing adverse effects on, and protecting the environment. It is supported by sustainable approaches for asset maintenance, integrity management, monitoring applications, health, and safety.

Vessel inspections, which are a substantial contributor to production downtime and corrosion under insulation, which is a common cause of abrupt shutdowns, are becoming more common–and implementing innovative solutions is becoming a need in many locations, particularly offshore. In a nutshell, the different aspects of asset management are the same for most offshore facilities. However, there may be some unique exceptions, such as under harsh weather conditions such as hurricanes, arctic environments, and seismic loads, which require a technical understanding of those environments and the behavior of the offshore structure under the worst-case scenario. However, the development and design of AIMS for offshore platforms and other offshore facilities helps to ensure that the platform is in use and can survive further, based on its approved extended life and structural integrity tests being passed as fit for use by the governing regulatory bodies such as ABS, BSEE, BSI, SON, DNV, and IMO. It is our opinion that these guidelines are solutions to improve asset monitoring to ensure that the offshore structures are better maintained, more durable, more reliable, and more sustainable. Further studies are recommended in the use of IoT (Internet of things), GIS (geographic information system) and AI (artificial intelligence) for automated remote asset monitoring of offshore facilities.

## Figures and Tables

**Table 1:** An example of a risk matrix that is applied in the offshore industry.

PoF						
CoF	1	2	3	4	5	6
**1**						
**2**						
**3**						
**4**						
**5**						
**6**						

**Table 2:** Sustainable maintenance management found on offshore structures that are among the longest standing fixed offshore platforms.

Platform	Installation Year	Operator	Location
Ekofisk 2/4 B Platform	1974	ConocoPhillips	70 m
Ekofisk 2/4 C Platform	1974	ConocoPhillips	70 m
Forties Alpha Platform	1975	Apache Corporation	95–128 m
Forties Charlie Platform	1975	Apache Corporation	95–128 m
Forties Bravo Platform	1975	Apache Corporation	95–128 m
Forties Delta Platform	1975	Apache Corporation	95–128 m
Cognac Drilling and Production Platform	1978	Shell	1025 m
Boubon Platform	1978	Fieldwood Energy	130 m
Statfjord A Platform	1979	Equinor	145 m
Eldfisk B Platform	1979	ConocoPhillips	70 m
Cerveza Platform	1981	Fieldwood SD	285 m
Ligera Platform	1982	Fieldwood SD	282 m
Statfjord B Platform	1982	Equinor Energy	70 m
Boxer Platform	1986	Whistler Energy II	229 m
Boxer Platform	1988	Shell	229 m

**Table 3:** Typical report for obsolescence risk (OR) category and recommended action. (Note: Green means active, Yellow means mature, Brown means retired and Red means end-of-life).

Obsolescence Risk (OR) Category & Recommended Action			
**OR Code**	**OR Category**	**OR Category Description**	Recommended Action
**A**	Active	The Original Equipment Manufacturer’s (OEM’s) current main product. The hardware and/or software are available and supported.	No additional action required.
**M**	Mature	Not the latest product. The hardware and/or software are available and supported.	It is recommended to review the assets expected lifetime spares requirement before the product goes to retired status. Ensure drawings, data sheets, manuals & specifications are kept and comprehensive. Ensure sufficient skills are maintained. Consider a replacement plan.
**R**	Retired	The hardware and/or software have limited support. A failed item would require exchange for a spare, repaired or re-manufactured item, or an equivalent.	It is recommended to review the risk and produce a strategic action plan before a failure affects business performance.
**E**	End of Life	The hardware and/or software are not sup- ported. Exchange part not available. Repair or Re-manufacture not possible.	It is recommended to review the risk and produce a strategic action plan before a failure affects business performance.
**S**	Site Survey	Not enough information to categorize it.	Site Survey Required.
**U**	Unobtainable information	Unable to contact supplier or unable to obtain information from supplier.	It is recommended to review the risk and determine action.
**N**	No OR	No OR category required because the part is a generic commercially available item.	No additional action required.
**O**	Outside OR	Outside standard OR life cycle. Item remains available, but would require remanufacturing.	Attention should be paid to manufacturing and delivery time constraints

**Table 4:** Patents on offshore monitoring systems, asset management, and structural integrity.

Patent	Publication Year	Assignee	Patent Title	Reference
US7194913	27 March 2007	Denby Grey Morrison; Jeremy R. Dean	Apparatus and methods for monitoring stress in steel catenary risers	[385]
US7328741	12 February 2008	John Allen; Antonio J. Pinto	System for sensing riser motion	[386]
US20050283276A1	18 May 2005	Clifford Prescott; David Brower	Real-time subsea monitoring and control system for pipelines	[389]
US7277162B2	2 October 2007	Jerry Gene Williams	Dynamic performance monitoring of long slender structures using optical fiber strain sensors	[390]
US5932815A.	3 August 1999	Donald J. Dodds	Anchor monitoring system.	[393]
US3579182A	18 May 1971	Leonard Schneider	Anchor dragging alarm control based on anchor inclination	[394]
US3722268A	27 March 1973	Global Marine Inc.	Load indicator for mooring line	[395]
US3913396A	21 October 1975	Secretary Trade Ind Brit	Monitoring system for moored floating vessels	[396]
US4258566A	31 March 1981	Decker Engineering Corporation	Load indicating apparatus having a hydraulic sensing unit and coupling pin-type electronic sensing unit	[397]
US4651139A	17 March 1987	Oettli Martin W.	Method for monitoring the drift of an anchored vessel and device for implementing the method	[398]
US20040035215A1	26 February 2004	Hanna Douglas S.	Load monitoring systems and methods	[399]
US20070175639A1	2 August 2007	Vetco Aibel As	Method and a device for monitoring and/or controlling a load on a tensioned elongated element	[400]
US20090115622A1	7 May 2009	Brian Edward Michie	Method of determining and monitoring a distance travelled by a marine vessel connected to anchor	[401]
US20100133843A1	3 June 2010	Hifunda, Llc	Method and device for harvesting energy from ocean waves	[402]
US20130279298A1	24 October 2013	William Mark Prentice	Monitoring of underwater mooring lines	[403]
US20180339753A1	29 November 2018	Fujitsu Limited	Alert control system, alert control method, and recording medium storing alert control program	[404]
US6543296B1	8 April 2003	Ricardo J. Bermudez	Method of monitoring/measuring rigging loads	[405]
US6901818B1	7 June 2005	Maxwell C. Cheung	Tension measuring device for mooring line	[406]
US20210115780A1	22 April 2021	Brendan Peter Hyland	Subsea structure monitoring system	[407]
US9228428B2	5 January 2016	General Electric Company	System and method for monitoring tubular components of a subsea structure	[408]
US10168253B2	1 January 2019	General Electric Company	Marine riser management system including subsea acoustic monitoring platform and an associated method	[409]
US11346744B2	31 May 2022	Nkt Hv Cables Ab	Method and system for fatigue-monitoring of a submarine cable in off-shore operations	[410]
US20050100414	12 May 2005	Mamdouh Salama	Composite riser with integrity monitoring apparatus and method	[411]

**Table 5:** List of some standards bodies and certification agencies.

Different National/International Standards Bodies and Certification Agencies	
International Organisation of Standardization (ISO)	Det Norske Veritas & Germaine Lloyds (DNV GL)
International Electrotechnical Commission (IEC)	International Civil Aviation Organization (ICAO)
Institute of Electrical and Electronics Engineers (IEEE)	American Society for Testing and Materials (ASTM)
Standards Organisation of Nigeria (SON)	Bureau of Indian Standards (BIS)
International Maritime Organization (IMO)	American Petroleum Institute (API)
Bureau Veritas (BV)	British Standards Institution (BSI)
European Standard (EN)	National Fire Protection Association, (NFPA)
American Society of Mechanical Engineers (ASME)	American National Standards Institute (ANSI)
International Association of Marine Aids to Navigation and Lighthouse Authorities (IALA)	Industry standards for the Norwegian continental shelf (NORSOK)
Standards Council of Canada (SCC)	Danish Standards Association (DSA)
Lloyds Registers	Standards Norway (NORSOK)
American Bureau of Shipping (ABS)	Bureau of Safety and Environmental Enforcement (BSEE)

**Table 6:** Standards related to asset management, integrity, reliability and monitoring.

Standard’s Reference	Title of Standard
BS ISO 55001:2014; ISO 55001:2014	Asset management—Management systems—Requirements
BS ISO 55002:2018; ISO 55002:2018	Asset management—Management systems—Guidelines for the application of ISO 55001
ASTM E2675-22	Standard Practice for Asset Management System Outcomes
ASTM E2812-17	Standard practice for uniform data management in asset management records systems
ASTM E3257-21	Standard practice for asset taxonomy.
ASTM E3035-15(2020)	Standard classification for facility asset component tracking system (FACTS).
PD IEC/TR 62978:2017	HVDC installations. Guidelines on asset management.
BS ISO 15686-6:2004	Buildings and constructed assets. Service life planning. Procedures for considering environmental impacts
BS 8536-2:2016	Briefing for design and construction. Code of practice for asset management (Linear and geographical infrastructure)
ASTM E2983-14(2019)	Standard guide for application of acoustic emission for structural health monitoring.
BS IEC/IEEE 80005-2:2016	Utility connections in port. High and low voltage shore connection systems. Data communication for monitoring and control
ASTM F3079-14(2020)	Standard practice for use of distributed optical fiber sensing systems for monitoring the impact of ground movements during tunnel and utility construction on existing underground utilities.
BS EN 13160-6:2016	Leak detection systems. Sensors in monitoring wells
BS EN ISO 17643:2015	Non-destructive testing of welds—Eddy current testing of welds by complex plane analysis.
ISO 15548-1	Non-destructive testing—Equipment for eddy current examination—Instrument characteristics and verification
ISO 15548-2	Non-destructive testing –Equipment for eddy current examination –Part 2: Probe characteristics and verification
BS EN 1711:2000	Non-destructive examination of welds. Eddy current examination of welds by complex plane analysis
ISO 15549:2008	Non-destructive testing—Eddy current testing—General principles
ISO 15548-3:2008	Non-destructive testing—Equipment for eddy current examination—Part 3: System characteristics and verification
ISO 19902	Petroleum and natural gas industries—Fixed steel offshore structures
ISO 16587:2004	Mechanical vibration and shock. Performance parameters for condition monitoring of structures.
BS EN ISO 18797-2:2021	Petroleum, petrochemical, and natural gas industries. External corrosion protection of risers by coatings and linings. Maintenance and field repair coatings for riser pipes
API RP 2SIM:2014	Structural integrity management of fixed offshore structures—recommended practice.
API RP 17N	Subsea production system reliability and technical risk management and integrity management
API RP 14J	Recommended Practice for Design and Hazards Analysis for Offshore Production Facilities
API RP 75	Recommended Practice for Development of a Safety and Environmental Management Program for Outer Continental Shelf (OCS) Operations and Facilities
API RP 581: 2016	Risk-Based Inspection Methodology
API RP 580: 2016	Risk-Based Inspection
API RP 574: 2016	Inspection Practices for Piping System Components
API RP 575: 2020	Inspection Practices for Atmospheric and Low Pressure Storage Tanks
API RP 754: 2021	Process Safety Performance Indicators for the Refining and Petrochemical Industries
API 570: 2016	Piping Inspection Code: In-service Inspection, Rating, Repair, and Alteration of Piping Systems
API 510	Pressure Vessel Inspection Code: Maintenance Inspection, Rating, Repair, and Alteration
API Standard 598	Valve Inspection and Testing
API RP 578	Material Verification Program for New and Existing Piping Systems
API RP 577	Welding Inspection and Metallurgy
API RP 576	Inspection of Pressure-relieving Devices
API RP 574	Inspection Practices for Piping System Components
API RP 583	Corrosion Under Insulation
API RP 584	Integrity Operating Windows
ASME CA-1:2020	Conformity assessment requirements
ASME PCC-1	Guidelines for Pressure Boundary Bolted Flange Joint Assembly
ASME PCC-2	Repair of Pressure Equipment and Piping
ISO 20815	Production assurance and reliability management
ISO 6385:2004	Ergonomic principles in the design of work systems
ISO Guide 73: 2009; ISO 31073:2022	Risk Management—Vocabulary.
ISO 31000:2018	Risk management—Guidelines
ISO 31000:2009	Risk Management—Principles and guidelines
IEC 31010; ISO 31010:2019	Risk Management—Risk Assessment Techniques.
ISO-22316:2017	Security and resilience—Organizational resilience—Principles and attributes
ISO 2394:2015	General Principles on Reliability for Structures
ISO 17776:2016	Petroleum and natural gas industries—Offshore production installations—Major accident hazard management during the design of new installations
ISO 19900	Petroleum and natural gas industries—General requirements for offshore structures
ISO/TR 31004:2013	Risk management—Guidance for the implementation of ISO 31000
NORSOK N-006:2015	Assessment of structural integrity for existing offshore load-bearing structures
NORSOK Z-013: 2010	Risk and emergency preparedness assessment
NORSOK N-005:2017	Condition monitoring of load bearing structures
NORSOK S-001	Technical safety
NORSOK S-002	Working environment
NORSOK N-004:2004	Design of steel structures
NORSOK Y-002:2010	Life Extension for Transportation Systems
ISO 19011	Guidelines for auditing management systems
ISO 9000	Quality management systems—Fundamentals and vocabulary
ISO 9001	Quality management systems—Requirements
ISO 14001	Environmental management systems
ISO 55000:2014	Asset management—What to do and why?
ISO 55000:2016	Asset management—Overview, principles, and terminology
ISO 14224:2016	Petroleum, petrochemical and natural gas industries—Collection and exchange of reliability and maintenance data for equipment
ISO/TS 12747:2011	Recommended Practice for Pipeline Life Extension
NACE RP 0472	Methods and Controls to Prevent In-Service Environmental Cracking of Carbon Steel Weldments in Corrosive Petroleum Refining Environments
NACE MR 0103	Materials Resistant to Sulfide Stress Cracking in Corrosive Petroleum Refining Environments
NACE SP 0102	In-Line Inspection of Pipelines
NACE RP 0502	Pipeline External Corrosion Direct Assessment. Methodology
IEC 61508-0	Functional safety for electrical, electronic and programmable electronic safety related systems
IEC 61508-1	General requirements
IEC 61508-2	Requirements for E/E/PE safety-related systems
IEC 61508-3	Software requirements
IEC 61508-4	Definitions and abbreviations
IEC 61508-5	Examples and methods for the determination of safety integrity levels
IEC 61508-6	Guidelines on the application of IEC 61508-2 and IEC 61508-3
IEC 61508-7	Overview of techniques and measures
IEC 61511	Process industries
IEC 61400-1 2010	Wind turbine. part 1: Design requirements
IEC 61400-4 2012	Wind turbines. part 4: Design requirements for wind turbine gearboxes
IEC 61400-3 2009	Wind turbines. part 3: Design requirements for offshore wind turbines
DNV RP-A203	Qualification procedures for new technology
DNV-RP-H101	Risk Management in Marine—and Subsea Operations
DNVGL-RP-C208 2016	Determination of Structural Capacity by Non-linear FE analysis Methods
DNV-CG-0121	Offshore Classification Based on Performance Criteria Determined from Risk Assessment Methodology
DNVGL-RP-G101	Risk-based inspection of offshore topsides static mechanical equipment
NFPA 704	Standard System for the Identification of the Hazards of Materials for Emergency Response

## Data Availability

The data for this study are not shared as it is an aspect of an on-going study in this present research.

## References

[1] Gallun R.A., Wright C.J., Nichols L.M., Stevenson J.W. (2001). Fundamentals of Oil and Gas Accounting.

[2] Bull A.S., Love M.S. (2019). Worldwide oil and gas platform decommissioning: A review of practices and reefing options. Ocean Coast. Manag..

[3] Kaiser M.J. (2021). A Review of Exploration, Development, and Production Cost Offshore Newfoundland. Nat. Resour. Res..

[4] Kaiser M.J., de Klerk A., Gary J.E., Handwerk G.E. (2020). Petroleum Refining: Technology, Economics, Markets.

[5] Kaiser M.J., Snyder B. (2013). Capital investment and operational decision making in the offshore contract drilling industry. Eng. Econ..

[6] Kaiser M.J., Pulsipher A.G. (2007). Generalized Functional Models for Drilling Cost Estimation. SPE J. Drill. Complet..

[7] Kaiser M.J., Narra S. (2019). An empirical evaluation of economic limits in the deepwater U.S. Gulf of Mexico. J. Nat. Gas Sci. Eng..

[8] D’Souza R.B., Shiladitya B. Field Development Planning and Floating Platform Concept Selection for Global Deepwater Developments. Proceedings of the Offshore Technology Conference.

[9] (2014). Asset Management—Overview, Principles and Terminology. 2014, International-Organization-for-Standardization. Technical Committee: ISO/TC 251 Asset Management.

[10] El-Reedy M. (2022). Asset Integrity Management for Offshore and Onshore Structures.

[11] Diop I., Abdul-Nour G., Komljenovic D. (2021). Overview of Strategic Approach to Asset Management and Decision-Making. Int. J. Eng. Res. Technol. (IJERT).

[12] Munn K., Goh S., Basson M., Thorpe D. (2021). Asset management competency requirements in Australian local government: A systematic literature review. Australas. J. Eng. Educ..

[13] Amadi-Echendu J., Willett R., Brown K., Hope T., Lee J., Mathew J., Vyas N., Yang B.S., Amadi-Echendu J., Brown K., Willett R., Mathew J. (2010). What Is Engineering Asset Management?. Definitions, Concepts and Scope of Engineering Asset Management. Engineering Asset Management Review.

[14] Mardiasmo D., Tywoniak S., Brown K., Burgess K. Asset Management and Governance—An Analysis of Fleet Management Process Issues in an Asset-Intensive Organization. Proceedings of the 1st International Conference on Infrastructure Systems and Services: Building Networks for a Brighter Future (INFRA 2008).

[15] Lohr C., Pena M. Stones Development: A Pioneering Management Philosophy for Enhancing Project Performance and Safety. Proceedings of the Offshore Technology Conference.

[16] Lohr C., Penney I. Stones Development: World Class Safety Performance in Singapore. Proceedings of the Offshore Technology Conference.

[17] Shahruddin T.S., Jenkins R.W., McFadyen M.K., Dechant S., Weber J.D. Kikeh Development: Project Overview. Proceedings of the Offshore Technology Conference.

[18] U.S. Coast Guard (1981). Collapse and Sinking of Mobile Offshore Drilling Unit Ranger I in the Gulf of Mexico on 10 May 1979 with Loss of Life: Marine Casualty Report.

[19] Whelan S. (2013). Petrobras P-36 Accident Rio de Janeiro, Brazil. J. Undergrad. Eng. Res. Scholarsh..

[20] NAP (2012). Macondo Well-Deepwater Horizon Blowout: Lessons for Improving Offshore Drilling Safety.

[21] USGov (2011). Deep Water: The Gulf Oil Disaster and the Future of Offshore Drilling (Report to the President).

[22] Cullen W.D. (1990). The Public Inquiry into the Piper Alpha Disaster: Volume 1.

[23] Cullen W.D. (1990). The Public Inquiry into the Piper Alpha Disaster: Volume 2.

[24] ConocoPhillips (2022). Emergency Preparedness.

[25] ConocoPhillips (2022). Health, Safety and Environment.

[26] ConocoPhillips (2022). HSE Management System.

[27] ConocoPhillips (2022). A Learning Organization.

[28] ConocoPhillips (2022). Process Safety.

[29] Templeton G., Konings S., Wilkie C., Benton P., Marcas G., McInally A., Rob I. Gryphon Field Development—Past, Present and Future. Proceedings of the SPE Offshore Europe Oil and Gas Conference and Exhibition.

[30] Ologun E.U., Wong K.Y., Chung Ee J.Y., Mammedov Y.D. (2022). Incorporating Sustainability and Maintenance for Performance Assessment of Offshore Oil and Gas Platforms: A Perspective. Sustainability.

[31] Garbie I.H., Al-Shaqsi R. (2019). Building sustainable models and assessments into petroleum companies: Theory and application. Int. J. Ind. Syst. Eng..

[32] Wan Mahmood W.H., Ab Rahman M.N., Deros B.M., Mazli H. (2011). Maintenance management system for upstream operations in oil and gas industry: A case study. Int. J. Ind. Syst. Eng..

[33] Moan T. (2005). Reliability-based management of inspection, maintenance and repair of offshore structures. Struct. Infrastruct. Eng..

[34] Moan T. (2018). Life cycle structural integrity management of offshore structures. Struct. Infrastruct. Eng..

[35] Moan T. (2017). Integrity management of offshore structures and its implication on computation of structural action effects and resistance. IOP Conf. Ser. Mater. Sci. Eng..

[36] Parker T.C., Sofidiya A. Erha and Erha North Development: Overview. Proceedings of the Offshore Technology Conference.

[37] Rui Z., Li C., Peng F., Ling K., Chen G., Zhou X., Chang H. (2017). Development of industry performance metrics for offshore oil and gas project. J. Nat. Gas. Sci. Eng..

[38] Frangopol D.M., Liu M. (2007). Maintenance and management of civil infrastructure based on condition, safety, optimization, and life-cycle cost. Struct. Infrastruct. Eng..

[39] Boutrot J., Legregeois N. Integrity Management Services for Floating Units from Design to Decommissioning. Proceedings of the Offshore Technology Conference.

[40] Rocher A., Perrollet C., Muir K. Asset Integrity Management—From General Requirements to Subsea Facilities: Total Block 17 Experience. Proceedings of the Offshore Technology Conference.

[41] Corcoran C., Stroubakis D. Asset Integrity Management—Raising the Bar on Safety. Proceedings of the SNAME 23rd Offshore Symposium.

[42] Adair S., Filmalter E., Mahlangu F. Asset Integrity Management in the Digital Age. Proceedings of the 19th World Petroleum Congress.

[43] Botto A., Rees J., Hull M. Holistic Approach to Subsea Integrity Management & Reliability and their Application to Greenfield and Brownfield Projects. Proceedings of the Offshore Technology Conference.

[44] Biasotto P., Rouhan A. Feedback from Experience on Structural Integrity of Floating Offshore Installations. Proceedings of the Offshore Technology Conference.

[45] Noli G., Fuggini C., Donisi D., Rossi A., Berardis S. Offshore Facilities Integrity Monitoring and Management. Proceedings of the Offshore Mediterranean Conference and Exhibition.

[46] Newman M.S.J., Reeder M.L., Woodruff A.H.W., Hatton I.R. (1993). The geology of the Gryphon Oil Field. Geol. Soc. Lond. Pet. Geol. Conf. Ser..

[47] Hashemi S.J., Javadpour S., Bajestani M.N., Zahiri M.R. Development and Application of Pressure Systems Integrity Management through Risk Based Inspection Audits in Abadan Refinery. Proceedings of the Abu Dhabi International Petroleum Exhibition and Conference.

[48] Hart N.J., Ageneau G., Hardie J. Development of the Gryphon Field Massive Injection Wing—Technical Challenges and Risks. Proceedings of the SPE Offshore Europe Oil and Gas Conference and Exhibition.

[49] Vinnem J.-E., Røed W. (2020). Offshore Risk Assessment, Volume 1: Principles, Modelling and Applications of QRA Studies.

[50] Vinnem J.-E. (2014). Offshore Risk Assessment, Volume 2: Principles, Modelling and Applications of QRA Studies.

[51] Hassel M., Utne I.B., Vinnem J.E. (2017). Allision risk analysis of offshore petroleum installations on the Norwegian Continental Shelf—An empirical study of vessel traffic patterns. WMU J. Marit. Affairs.

[52] Poulassichidis T. Application of Risk Based Inspection to Offshore Facilities. Proceedings of the SPE Annual Technical Conference and Exhibition.

[53] Reynolds J.T. Risk Based Inspection—Where Are We Today? In Proceedings of the CORROSION 2000. https://onepetro.org/NACECORR/proceedings-abstract/CORR00/All-CORR00/NACE-00690/111761.

[54] Areeniyom P. The Use of Risk-Based Inspection for Aging Pipelines in Sirikit Oilfield. Proceedings of the International Petroleum Technology Conference.

[55] Dewanto K., Christian R., Wibowo R. Development and Implementation of Risk Based Inspection Methodology in Managing Inspection of Pressurized Production Facilities. Proceedings of the SPE Asia Pacific Conference on Integrated Modelling for Asset Management.

[56] Clement D.L., Soemarman W., Sulistiyono M. Business Integration of Safety, Health and Environmental Management. Proceedings of the SPE Health, Safety and Environment in Oil and Gas Exploration and Production Conference.

[57] Lamki A.M.N., Binks S.L.M. Application of HSE Management in a Multi-Cultural Environment. Proceedings of the SPE Health, Safety and Environment in Oil and Gas Exploration and Production Conference.

[58] Visser J.P. Managing Safety in the Oil Industry—The Way Ahead. Proceedings of the 14th World Petroleum Congress.

[59] Downey I.L. E & P FORUM Health, Safety and Environmental Management System Guidelines. Proceedings of the SPE Offshore Europe.

[60] Al-Failakawi A.H., Bala S.S.A. Business Partners’ Safety: Obligation or Opportunity? In Proceedings of the ASSE Professional Development Conference and Exposition.

[61] Doherty B.D., Fragu L.P. Sustainable HSE Performance: Successful Management Systems and Monitoring Tools in the Middle East LNG Industry. Proceedings of the SPE International Conference on Health, Safety and Environment in Oil and Gas Exploration and Production.

[62] Walters K.W., Wallace J. Moving Beyond Management System Descriptions to Achieve a Step Change in HSE Performance. Proceedings of the SPE International Health, Safety & Environment Conference.

[63] Doulabi H., Khamseh A., Torabi T. (2020). A System Dynamics Approach to Designing Technological Innovation Management Model in Downstream Petrochemical Industries. J. Syst. Manag..

[64] Vijayalakshmi B.S. (2016). Development of Sustainable Production Indicators Using the Analytical Hierarchy Process for the Petrochemical Industry in Malaysia. Ph.D. Thesis.

[65] Samuel V.B., Agamuthu P., Hashim M. (2013). Indicators for assessment of sustainable production: A case study of the petrochemical industry in Malaysia. Ecol. Indic..

[66] Aryanasl A., Ghodousi J., Arjmandi R., Mansouri N. (2017). Components of sustainability considerations in management of petrochemical industries. Environ. Monit. Assess..

[67] Sari E., Shaharoun A.M., Ma’aram A., Mohd Yazid A. (2015). Sustainable maintenance performance measures: A pilot survey in Malaysian automotive companies. Proceedings of the Procedia CIRP.

[68] Seidel S., Recker J.C., Pimmer C., vom Brocke J. Enablers and barriers to the organizational adoption of sustainable business practices. Proceedings of the 16th Americas Conference on Information Systems: Sustainable IT Collaboration around the Globe.

[69] Muchiri P., Pintelon L., Gelders L., Martin H. (2011). Development of maintenance function performance measurement framework and indicators. Int. J. Prod. Econ..

[70] (2016). Najafi, M. Pipeline Infrastructure Renewal and Asset Management.

[71] Frangopol D.M. (2011). Life-cycle performance, management, and optimisation of structural systems under uncertainty: Accomplishments and challenges. Struct. Infrastruct. Eng..

[72] Drożyner P. (2021). The impact of the implementation of management system on the perception of role and tasks of maintenance services and effectiveness of their functioning. J. Qual. Maint. Eng..

[73] Moldan B., Janoušková S., Hák T. (2012). How to understand and measure environmental sustainability: Indicators and targets. Ecol. Indic..

[74] Mascarenhas A., Coelho P., Subtil E., Ramos T.B. (2010). The role of common local indicators in regional sustainability assessment. Ecol. Indic..

[75] Fernandez-Sanchez G., Rodriquez-Lopez F. (2010). A methodology to identify sustainability indicators in construction project management application to infrastructure projects in Spain. Ecol. Indic..

[76] Almeida CM V.B., Agostinho F., Giannetti B.F., Huisingh D. (2015). Integrating cleaner production into sustainability strategies: An introduction to this special volume. J. Clean. Prod..

[77] Ling F.Y.Y., Low S.P., Wang S.Q., Lim H.H. (2009). Key project management practices affecting Singaporean firms’ project performance in China. Int. J. Proj. Manag..

[78] Luu V.T., Kim S.-Y., Huynh T.-A. (2008). Improving project management performance of large contractors using benchmarking approach. Int. J. Proj. Manag..

[79] Rui Z., Peng F., Ling K., Chang H., Chen G., Zhou X. (2017). Investigation into the performance of oil and gas projects. J. Nat. Gas Sci. Eng..

[80] Yun S., Choi J., de Oliveira D.P., Mulva S.P. (2016). Development of performance metrics for phase-based capital project benchmarking. Int. J. Proj. Manag..

[81] Chakrabarti S.K. (2005). Handbook of Offshore Engineering.

[82] El-Reedy M. (2012). Offshore Structures: Design, Construction and Maintenance.

[83] Bai Y., Bai Q. (2010). Subsea Engineering Handbook.

[84] Wilson J. (2002). Dynamics of Offshore Structures.

[85] Chandrasekaran S. (2018). Dynamic Analysis and Design of Offshore Structures.

[86] Barltrop N.D.P., Adams A.J. (1991). Dynamics of Fixed Marine Structures.

[87] Offshore Technology (2019). The Longest Standing Fixed Offshore Platforms.

[88] Brebbia C.A., Walker S. (1979). Dynamic Analysis of Offshore Structures.

[89] Leffler W.L., Pattarozzi R., Sterling G. (2011). Deepwater Petroleum Exploration & Production: A Nontechnical Guide.

[90] Fang H., Duan M. (2014). Offshore Operation Facilities.

[91] Aird P. (2019). Deepwater Drilling: Well Planning, Design, Engineering, Operations, and Technology Application.

[92] Samie N.N. (2016). Practical Engineering Management of Offshore Oil and Gas Platforms.

[93] Clews R.J. (2016). Project Finance for the International Petroleum Industry.

[94] Chandrasekaran S., Jain A.K. (2016). Ocean Structures, Construction, Materials, and Operations.

[95] Laik S. (2018). Offshore Petroleum Drilling and Production.

[96] Speight J.G. (2011). Handbook of Offshore Oil and Gas Operations.

[97] Primrose M.J., Bentley P.D., van der Graaf G.C., Sykes R.M. The HSE Management System in Practice-lmplementation. Proceedings of the SPE Health, Safety and Environment in Oil and Gas Exploration and Production Conference.

[98] Madkour A.A. Operating Company’s (HSE) Management System (Guidelines, Practices & Results). Proceedings of the SPE International Conference on Health, Safety and Environment in Oil and Gas Exploration and Production.

[99] Weldon D., Wallace J. The What and How for HSE Management Systems. Proceedings of the SPE International Health, Safety & Environment Conference.

[100] Gibson D.W. Enhanced Environmental Management for Land Based Seismic Acquisition using a Quality, Health, Safety and Environmental Management System. Proceedings of the SPE International Conference on Health, Safety, and Environment in Oil and Gas Exploration and Production.

[101] Onianwa A.T., Onwuzurike-azu C., Fowler A., Pagett R., Ogunnaike B. Development and Implementation of HSE Management System (HSE-MS) in a Deepwater Company—Shell Nigeria Exploration & Production Company (SNEPCO) Experience. Proceedings of the SPE International Conference on Health, Safety and Environment in Oil and Gas Exploration and Production.

[102] Leuterman A.J.J., Candler J.E., Ceron R., Rabke S. Improving Environmental and Occupational Health Performance through a HSE Management System—A Seven Year Case Study. Proceedings of the SPE International Conference on Health, Safety, and Environment in Oil and Gas Exploration and Production.

[103] Campbell H., Polo J., Guillaume B. HSE Management System: Keep It Simple! In Proceedings of the International Conference on Health, Safety and Environment in Oil and Gas Exploration and Production.

[104] Macini P., Mesini E. (2018). History of petroleum and petroleum engineering. Petroleum Engineering—Upstream.

[105] Kontorovich A.E., Eder L.V., Filimonova V., Mishenin M.V., Nemov V.Y. (2016). Oil industry of major historical centre of the Volga-Ural petroleum province: Past, current state, and long-run prospects. Russ. Geol. Geophys..

[106] Udosen C., Etok A.S., George I.N. (2009). Fifty Years Of Oil Exploration In Nigeria: The Paradox Of Plenty. Glob. J. Soc. Sci..

[107] Krzywiec P., Craig J., Gerali F., MacAulay F., Sorkhabi R. (2018). The birth and development of the oil and gas industry in the Northern Carpathians (up until 1939). History of the European Oil and Gas Industry.

[108] Spencer A., Chew K. (2009). Petroleum exploration history: Discovery pattern versus manpower, technology and the development of exploration principles. First Break.

[109] Li J.-F., Ye J.-L., Qin X.-W., Qiu H.-J., Wu N.-Y., Lu H.-L., Xie W.-W., Lu J.-A., Peng F., Xu Z.-Q. (2018). The first offshore natural gas hydrate production test in South China Sea. China Geol..

[110] Craig J., Gerali F., Macaulay F., Sorkhabi R., Craig J., Gerali F., Macaulay F., Sorkhabi R. (2018). The history of the European oil and gas industry (1600s–2000s). History of the European Oil and Gas Industry.

[111] Craig J., Sorkhabi R. (2021). History of Oil: The Birth of the Modern Oil Industry (1859–1939). Encyclopedia of Petroleum Geoscience.

[112] Craig J., Sorkhabi R. (2021). Drilling: History of Onshore Drilling and Technology. Encyclopedia of Petroleum Geoscience.

[113] Craig J., Sorkhabi R. (2021). History of Oil: The Premodern Era (Thirteenth to Mid-Nineteenth Centuries). Encyclopedia of Petroleum Geoscience.

[114] Craig J., Sorkhabi R. (2020). Barrel. Encyclopedia of Petroleum Geoscience.

[115] Craig J., Sorkhabi R. (2021). History of Oil: Regions and Uses of Petroleum in the Classical and Medieval Periods. Encyclopedia of Petroleum Geoscience.

[116] Glennie K.W., Ziegler K., Turner P., Daines S.R. (1997). History of exploration in the southern North Sea. Petroleum Geology of the Southern North Sea: Future Potential. Special Publications 123.

[117] Kaiser M.J. (2020). Offshore oil and gas records circa 2020. Ships Offshore Struct..

[118] Kaiser M.J. (2016). A review of deepwater pipeline construction in the U.S. Gulf of Mexico–Contracts, cost, and installation methods. J. Mar. Sci. Appl..

[119] Haritos N. (2007). Introduction to the analysis and design of offshore structures—An overview. Electron. J. Struct. Eng. (eJSE).

[120] Yu L.C., King L.S., Hoon A.T.C., Yean P.C.C. (2015). A Review Study of Oil and Gas Facilities for Fixed and Floating Offshore Platforms. Res. J. Appl. Sci. Eng. Technol..

[121] Amiri N., Shaterabadi M., Reza Kashyzadeh K., Chizari M. (2021). A Comprehensive Review on Design, Monitoring, and Failure in Fixed Offshore Platforms. J. Mar. Sci. Eng..

[122] Al-Sharif A.A. Design, fabrication and installation of fixed offshore platforms in the Arabian Gulf. Proceedings of the Fourth Saudi Engineering Conference, Saudi Arabian Oil Company.

[123] Ladeira I., Márquez L., Echeverry S., Le Sourne H., Rigo P. (2022). Review of methods to assess the structural response of offshore wind turbines subjected to ship impacts. Ships Offshore Struct..

[124] Saiful Islam A.B.M., Jameel M., Jumaat M.Z., Shirazi S., Salman F.A. (2012). Review of offshore energy in Malaysia and floating Spar platform for sustainable exploration. Renew. Sustain. Energy Rev..

[125] Al-Yafei E.F. (2018). Sustainable Design for Offshore Oil and Gas Platforms: A Conceptual Framework for Topside Facilities Projects. Ph.D. Thesis.

[126] Kreidler T.D. (1997). The Offshore Petroleum Industry: The Formative Years, 1945–1962. Ph.D. Thesis.

[127] Cavendish W. (2021). Arup Integrity Management: A Digital Approach for Safe and Effective Integrity Management.

[128] Sadeghi K. (2008). Significant guidance for design and construction of marine and offshore structures. GAU J. Soc. Appl. Sci..

[129] Sadeghi K., Dilek H. (2019). An Introduction to the design of Offshore Structures. Acad. Res. Int..

[130] Khan R., Mad A.B., Osman K., Aziz M.A.A., Márquez F.P.G., Papaelias M. (2019). Maintenance Management of Aging Oil and Gas Facilities. Maintenance Management.

[131] Gowda S.S., Hassinen P. (1992). Development of Offshore Structures—An Overview. IABSE Congress Report {Rapport du Congrès AIPC = IVBH Kongressbericht}, 14(1992). https://www.e-periodica.ch/cntmng?pid=bse-cr-002%3A1992%3A14%3A%3A43.

[132] Kosleck S., Clauss G.F., Lee Y.J. (2004). Deepwater Solutions for Offshore Production Technology (Offshore-Förderplattformen: Entwicklungen für die Tiefsee). Proceedings of the Annual General Conference of the German Society for Maritime Technology.

[133] Walker S., Tarantola S. (2018). Guidelines for Inspections of Offshore Installations.

[134] Yew W.K., Ismail S., Sabri H.A.R., Rahim A.R.A. Project Management of Oil and Gas Project in Malaysia. Proceedings of the International Association for Asia Pacific Studies 5th Annual Conference.

[135] Ronalds B.F. (2005). Applicability ranges for offshore oil and gas production facilities. Mar. Struct..

[136] Reddy D., Swamidas A. (2013). Essentials of Offshore Structures: Theory and Applications.

[137] Wang C.M., Utsunomiya T., Wee S.C., Choo Y.S. (2010). Research on floating wind turbines: A literature survey. IES J. Part A Civ. Struct. Eng..

[138] Mustapa M.A., Yaakob O.B., Ahmed Y.M., Rheem C.-K., Koh K.K., Adnan F.A. (2017). Wave energy device and breakwater integration: A review. Renew. Sustain. Energy Rev..

[139] Bai Y., Bai Q. (2018). Subsea Engineering Handbook.

[140] Muyiwa O.A., Sadeghi K. (2007). Construction planning of an offshore petroleum platform. GAU J. Soc. Appl. Sci..

[141] Holmes T., Connolly S., Wilday J., Hare J., Walsh P. Managing fire and explosion hazards on offshore ageing installations. Proceedings of the IChemE Symposium Series No. 155, Hazards XXI.

[142] Sadeghi K. (2007). An Overview of Design, Analysis, Construction and Installation of Offshore Petroleum Platforms Suitable for Cyprus Oil/Gas Fields. GAU J. Soc. Appl. Sci..

[143] Chandrasekaran S., Gaurav S. (2017). Design Aids for Offshore Structures under Special Environmental Loads, Including Fire Resistance.

[144] Amaechi C.V., Chesterton C., Butler H.O., Wang F., Ye J. (2021). Review on the design and mechanics of bonded marine hoses for Catenary Anchor Leg Mooring (CALM) buoys. Ocean Eng..

[145] Amaechi C.V., Chesterton C., Butler H.O., Wang F., Ye J. (2021). An Overview on Bonded Marine Hoses for sustainable fluid transfer and (un)loading operations via Floating Offshore Structures (FOS). J. Mar. Sci. Eng..

[146] Amaechi C.V., Wang F., Ja’E I.A., Aboshio A., Odijie A.C., Ye J. (2022). A literature review on the technologies of bonded hoses for marine applications. Ships Offshore Struct..

[147] Amaechi C.V., Chesterton C., Butler H.O., Gillet N., Wang C., Ja’E I.A., Reda A., Odijie A.C. (2022). Review of Composite Marine Risers for Deep-Water Applications: Design, Development and Mechanics. J. Compos. Sci..

[148] Toh W., Bin Tan L., Jaiman R.K., Tay T.E., Tan V.B.C. (2018). A comprehensive study on composite risers: Material solution, local end fitting design and global response. Mar. Struct..

[149] Pham D.-C., Sridhar N., Qian X., Sobey A.J., Achintha M., Shenoi A. (2016). A review on design, manufacture and mechanics of composite risers. Ocean Eng..

[150] Ochoa O.O., Salama M.M. (2005). Offshore composites: Transition barriers to an enabling technology. Compos. Sci. Technol..

[151] Anastasiades K., Michels S., Van Wuytswinkel H., Blom J., Audenaert A. (2022). Barriers for the circular reuse of steel in the Belgian construction sector: An industry-wide perspective. Proc. Inst. Civ. Eng.—Manag. Procure. Law.

[152] Dareing D.W. (2012). Mechanics of Drillstrings and Marine Risers.

[153] Sparks C. (2018). Fundamentals of Marine Riser Mechanics: Basic Principles and Simplified Analyses.

[154] Bai Y., Bai Q. (2005). Subsea Pipelines and Risers.

[155] Bai Y., Bai Q., Ruan W. Flexible Pipes: Advances in Pipes and Pipelines.

[156] Sævik S. (1992). On Stresses and Fatigue in Flexible Pipes. Ph.D. Thesis.

[157] Amaechi C.V. (2022). Novel Design, Hydrodynamics and Mechanics of Marine Hoses in Oil/Gas Applications. Ph.D. Thesis.

[158] Byrom T.G. (2015). Casing and Liners for Drilling and Completion: Design and Application. A Volume in Gulf Drilling Guides.

[159] Grace R.D. (2017). Blowout and Well Control Handbook.

[160] Joshi S.D. (1991). Horizontal Well Technology.

[161] Stewart G. (2011). Well Test Design and Analysis.

[162] Azar J.J., Samuel R. (2007). Drilling Engineering.

[163] Renpu W. (2011). Advanced Well Completion Engineering.

[164] Caenn R., Darley H.C.H., Gray G.R. (2017). Composition and Properties of Drilling and Completion Fluids.

[165] Devereux S. (1998). Practical Well Planning and Drilling Manual.

[166] Veatch R.W., King G.E., Holditch S.A. (2017). Essentials of Hydraulic Fracturing: Vertical and Horizontal Wellbores.

[167] Raymond M.S., Leffler W.L. (2017). Oil & Gas Production in Nontechnical Language.

[168] Crumpton H. (2017). Well Control for Completions and Interventions.

[169] Amaechi C.V., Reda A., Butler H.O., Ja’E I.A., An C. (2022). Review on Fixed and Floating Offshore Structures. Part I: Types of Platforms with Some Applications. J. Mar. Sci. Eng..

[170] Amaechi C.V., Reda A., Butler H.O., Ja’E I.A., An C. (2022). Review on Fixed and Floating Offshore Structures. Part II: Sustainable Design Approaches and Project Management. J. Mar. Sci. Eng..

[171] Amaechi C.V., Reda A., Ja’E I.A., Wang C., An C. (2022). Guidelines on Composite Flexible Risers: Monitoring Techniques and Design Approaches. Energies.

[172] Soares C.G., Garbatov Y. (1999). Reliability of maintained ship hulls subjected to corrosion and fatigue under combined loading. J. Constr. Steel Res..

[173] Soares C.G., Garbatov Y. (1996). Fatigue reliability of the ship hull girder accounting for inspection and repair. Reliab. Eng. Syst. Saf..

[174] Hussein A., Soares C.G. (2009). Reliability and residual strength of double hull tankers designed according to the new IACS common structural rules. Ocean Eng..

[175] Chojaczyk A.A., Teixeira A.P., Neves L.C., Cardoso J.B., Soares C.G. (2015). Review and application of Artificial Neural Networks models in reliability analysis of steel structures. Struct. Saf..

[176] Gaspar B., Teixeira A.P., Soares C.G. (2014). Assessment of the efficiency of Kriging surrogate models for structural reliability analysis. Probabilistic Eng. Mech..

[177] Soares C.G., Garbatov Y. (1999). Reliability of maintained, corrosion protected plates subjected to non-linear corrosion and compressive loads. Mar. Struct..

[178] Teixeira A.P., Soares C.G., Netto T.A., Estefen S.F. (2008). Reliability of pipelines with corrosion defects. Int. J. Press. Vessel. Pip..

[179] Aboshio A., Uche A.O., Akagwu P., Ye J. (2021). Reliability-based design assessment of offshore inflatable barrier structures made of fibre-reinforced composites. Ocean Eng..

[180] Fischer K.P., Bue B.P., Brattas L.P., Steensland O.P. Norwegian Continental Shelf North Of 62° N: Environmental Conditions and Corrosion Control. Proceedings of the Offshore Technology Conference.

[181] Weeter R.F. Downhole Corrosion—Prevention and Treatment. Proceedings of the International Petroleum Exhibition and Technical Symposium.

[182] Winning I.G., Taylor A., Ronceray M. Corrosion Mitigation—The Corrosion Engineers Options. Proceedings of the SPE International Conference on Oilfield Corrosion.

[183] Sun H., Blumer D.J., Swidzinski M., Davis J. Evaluating Corrosion Inhibitors for Sour Gas Subsea Pipelines. Proceedings of the International Petroleum Technology Conference.

[184] Amani M., Dawood H. A Comprehensive Review of Corrosion and its Inhibition in the Oil and Gas Industry. Proceedings of the SPE Kuwait Oil and Gas Show and Conference.

[185] Landers H.D. The economics of corrosion control in oil and gas production. Proceedings of the 6th World Petroleum Congress.

[186] Rippon I.J., Pots B.F., Girgis M., Goerz K. Failure Modes and Effect Analysis of a Sour Corrosion Control System. Proceedings of the CORROSION 2006.

[187] Norsworthy R. NACE Criteria and Effective Corrosion Control of External Corrosion on Pipelines. Proceedings of the CORROSION 2009.

[188] Norsworthy R. Causes of External Corrosion on Coated and Cathodically Protected Pipelines. Proceedings of the CORROSION 2009.

[189] HSE (2012). Ageing Plant and Life Extension.

[190] HSE (2020). Offshore Statistics & Regulatory Activity Report 2020.

[191] HSE (2019). Offshore Statistics & Regulatory Activity Report 2019.

[192] HSE (2017). Offshore Statistics & Regulatory Activity Report 2018.

[193] HSE (2011). Offshore safety statistics bulletin 2010/11.

[194] Hazardex (2020). HSE UK Offshore Safety Report for 2018. Hazardex. https://www.hazardexonthenet.net/article/177200/HSE-UK-offshore-safety-report-for-2018.aspx.

[195] HSE (2022). Effect on RIDDOR Statistics Following Recent Legal and System Changes.

[196] Yeter B., Garbatov Y. (2021). Optimal Life Extension Management of Offshore Wind Farms Based on the Modern Portfolio Theory. Oceans.

[197] Piel J.H., Stetter C., Heumann M., Westbomke M., Breitner M.H. (2013). Lifetime Extension, Repowering or Decommissioning? Decision Support for Operators of Ageing Wind Turbines. IOP Conf. Ser. J. Phys. Conf. Ser..

[198] Pakenham B., Ermakova A., Mehmanparast A. (2021). A Review of Life Extension Strategies for Offshore Wind Farms Using Techno-Economic Assessments. Energies.

[199] Ziegler L., Gonzalez E., Rubert T., Smolka U., Melero J.J. (2018). Lifetime extension of onshore wind turbines: A review covering Germany, Spain, Denmark, and the UK. Renew. Sustain. Energy Rev..

[200] Ziegler L., Lange J., Smolka U., Muskulus M. The decision on the time to switch from lifetime extension to repowering. Proceedings of the Wind Europe Summit 2016.

[201] Rubert T., McMillan D., Niewczas P. (2018). A decision support tool to assist with lifetime extension of wind turbines. Renew. Energy.

[202] Rubert T., Niewczas P., McMillan D. Life extension for wind turbine structures and foundations. Proceedings of the International Conference on Offshore Renewable Energy 2016.

[203] Casey J. (2020). Asset Life Extension: Viable in the Long Term for Oil and Gas?.

[204] Schumacher C., Weber F. How to extend the lifetime of wind turbines. *Renewable Energy World*, 20 September 2019. https://www.renewableenergyworld.com/2019/09/20/how-to-extend-the-lifetime-of-wind-turbines/.

[205] Astolfi D., Byrne R., Castellani F. (2021). Estimation of the Performance Aging of the Vestas V52 Wind Turbine through Comparative Test Case Analysis. Energies.

[206] Astolfi D., Byrne R., Castellani F. (2020). Analysis of Wind Turbine Aging through Operation Curves. Energies.

[207] Siemens (2014). Life Extension Program.

[208] OGUK (2014). Guidelines on the Management of Ageing and Life Extension of Offshore Structures—Issue 1 April 2012.

[209] OGUK (2012). Guidelines on the Management of Ageing and Life Extension for UKCS Oil and Gas Installations—Issue 1 April 2012.

[210] EI (2009). A Framework for Monitoring the Management of Ageing Effects on Safety Critical Elements. EI Research Report.

[211] EI (2022). Performance Standards for Structural Safety Critical Elements.

[212] EI (2020). Guidelines for Management of Safety Critical Elements (SCEs).

[213] EI (2007). Guidelines for the Management of Safety Critical Elements.

[214] EI (2020). Guidelines for the Identification and Management of Environmental Barriers.

[215] EI (2017). Guidelines on the Corrosion Management of Offshore Oil and Gas Production Facilities: Addressing Asset Ageing and Life Extension (ALE).

[216] DNV (2021). DNV-CG-0121 Offshore Classification Based on Performance Criteria Determined from Risk Assessment Methodology.

[217] (2010). NORSOK Y-002:2010; Life Extension for Transportation Systems.

[218] (2011). Recommended Practice for Pipeline Life Extension.

[219] (2015). Petroleum and Natural Gas Industries—Control and Mitigation of Fires and Explosions on Offshore Production Installations—Requirements and Guidelines.

[220] (2022). Risk Management—Vocabulary.

[221] HSE (2007). Key Programme 3—Asset Integrity Programme. A Report by the Offshore Division of HSE’s Hazardous Installations Directorate.

[222] HSE (2012). Key Programme 4 (KP4): Ageing and Life Extension Programme. Executive Summary. A Report by the Energy Division of HSE’s Hazardous Installations Directorate. November 2012.

[223] HSE (2006). Plant Ageing: Management of Equipment Containing Hazardous Fluids or Pressure.

[224] HSE (2010). Plant Ageing Study: Phase 1 Report.

[225] HSE (1999). Reducing Error and Influencing Behaviour.

[226] Dalzell G., Roberts T.A., Jagger S., Walsh P. (2007). Guidance on Fire and Explosion Hazards Associated with Ageing Offshore Oil and Gas Platforms.

[227] Chang K.C., Kuo P.T., Hsu K.R., Rao K.R. (2009). License Renewal and Aging Management. Companion Guide to the ASME Boiler and Pressure Vessel Code.

[228] Brkić D., Praks P. (2021). Probability Analysis and Prevention of Offshore Oil and Gas Accidents: Fire as a Cause and a Consequence. Fire.

[229] HSE (2009). Guidance of Management of Ageing and Thorough Reviews of Ageing Installations.

[230] HMSO (1995). The Offshore Installations (Prevention of Fire and Explosions, and Emergency Response) Regulations 1995.

[231] HMSO (2005). The Offshore Installations (Safety Case) Regulations 2005.

[232] Walker S., Konstantinidou M., Contini S., Zhovtyak E., Tarantola S. (2017). Guidance for the Assessment of Reports on Major Hazards Based on the Requirements of Directive 2013/30/EU—Summary and Highlights of the JRC Training Course under the Virtual Centre of Offshore Safety Expertise.

[233] HSE (2006). Guidance for the Topic Assessment of the Major Accident Hazard Aspects of Safety Cases.

[234] HSE (2021). Assessment Principles for Offshore Safety Cases (APOSC): Offshore Major Accident Regulator.

[235] HSE (2006). Assessment principles for offshore safety cases (APOSC).

[236] HSE (2006). Guidance on Risk Assessment for Offshore Installations.

[237] Sharif A., Aloui C., Yarovaya L. (2020). COVID-19 pandemic, oil prices, stock market, geopolitical risk and policy uncertainty nexus in the US economy: Fresh evidence from the wavelet-based approach. Int. Rev. Financ. Anal..

[238] Engebretsen R., Anderson C. (2020). The Impact of Coronavirus (COVID-19) and the Global Oil Price Shock on the Fiscal Position of Oil-Exporting Developing Countries.

[239] Kelly S. Oil Price Crashes into Negative Territory for the First Time in History Amid Pandemic. Reuters. 20 April 2020. https://reut.rs/35Pq49T.

[240] Millefiori L.M., Braca P., Zissis D., Spiliopoulos G., Marano S., Willett P.K., Carniel S. (2021). COVID-19 impact on global maritime mobility. Sci. Rep..

[241] Moriarty L.F., Plucinski M., Marston B.J., Kurbatova E.V., Knust B., Murray E.L., Pesik N., Rose D., Fitter D., Kobayashi M. (2020). Public Health Responses to COVID-19 Outbreaks on Cruise Ships—Worldwide, February–March 2020. Morb. Mortal. Wkly. Rep..

[242] Sackey A.D., Tchouangeup B., Lamptey B.L., van der Merwe B., Lee R.O.-D., Mensah R., Fuseini M.C., Sackey A.D. (2021). Outlining the challenges of Covid-19 health crises in Africa’s maritime industry: The case of maritime operations in marine warranty surveying practice. Marit. Stud..

[243] Lerche J., Lorentzen S., Enevoldsen P., Neve H. (2022). The impact of COVID -19 on offshore wind project productivity—A case study. Renew. Sustain. Energy Rev..

[244] Depellegrin D., Bastianini M., Fadini A., Menegon S. (2020). The effects of COVID-19 induced lockdown measures on maritime settings of a coastal region. Sci. Total Environ..

[245] Olukolajo M.A., Oyetunji A.K., Oluleye I.B. (2021). Covid-19 protocols: Assessing construction site workers compliance. J. Eng. Des. Technol..

[246] Amaechi C.V., Amaechi E.C., Amechi S.C., Oyetunji A.K., Kgosiemang I.M., Mgbeoji O.J., Ojo A.S., Abelenda A.M., Milad M., Adelusi I. (2022). Management of Biohazards and Pandemics: COVID-19 and Its Implications in the Construction Sector. Comput. Water Energy Environ. Eng..

[247] Shih W.C. Global Supply Chains in a Post-Pandemic World. Harvard Business Review, September–October 2020. https://hbr.org/2020/09/global-supply-chains-in-a-post-pandemic-world.

[248] Werner R., Jan G., Hannes G., Jürgen E., Nico P. (2021). Post COVID-19 Value Chains: Options for Reshoring Production Back to Europe in a Globalised Economy.

[249] Candina J., Fernández D.G., Hall S., Verre F. Reinventing Upstream Oil and Gas Operations after the COVID-19 Crisis. McKinsey & Company, 20 August 2020. https://www.mckinsey.com/industries/oil-and-gas/our-insights/reinventing-upstream-oil-and-gas-operations-after-the-covid-19-crisis.

[250] Barbosa F., Bresciani G., Graham P., Nyquist S., Yanosek K. (2020). Oil and Gas after COVID-19: The Day of Reckoning or a New Age of Opportunity?.

[251] Tang C.-S., Paleologos E.K., Vitone C., Du Y.-J., Li J.-S., Jiang N.-J., Deng Y.-F., Chu J., Shen Z., Koda E. (2021). Environmental geotechnics: Challenges and opportunities in the post-COVID-19 world. Environ. Geotech..

[252] Bouri E., Demirer R., Gupta R., Pierdzioch C. (2020). Infectious Diseases, Market Uncertainty and Oil Market Volatility. Energies.

[253] Agomuoh A.E., Ossia C.V., Chukwuma F.O. (2021). Asset Integrity Management in Mitigating Oil and Gas Pipeline Vandalism in the Niger Delta Region—Deep Burial Solution. World J. Eng. Technol..

[254] IQ (2019). What Is Asset Integrity? Oil and Gas IQ. https://www.oilandgasiq.com/oil-gas/news/what-is-asset-integrity.

[255] HSE (2015). Managing Health and Safety in Construction: Construction (Design and Management) Regulations 2015. Guidance on Regulations. Series L153.

[256] HSE (2006). Health and Safety in Construction. A Report by the Offshore Division of HSE’s Hazardous Installations Directorate. Series HSG150.

[257] Amaechi C.V. (2016). Health, Safety and Biohazards in Construction: How Safe Is the Work Place? Risks and Hazards in Our Environment.

[258] Comer P.J., Eades M.J. The Application of Risk Assessment in Offshore Projects. Proceedings of the Offshore Technology Conference.

[259] Kian-Hua S., Su-Fook L., Yun-Chung L. ALARP Demonstration in UCG Trial Production Operations (TPO) Facility Design. Proceedings of the International Petroleum Technology Conference.

[260] Wiig E., Berthelsen I., Donovan K. The Troll HSE Risk Management System. Proceedings of the SPE Health, Safety and Environment in Oil and Gas Exploration and Production Conference.

[261] Chetrit A. Major Risks Analysis with a Scenario Based Methodology. Proceedings of the SPE International Conference on Health, Safety, and Environment in Oil and Gas Exploration and Production.

[262] Whiting J.F. Effective Risk Assessment in TA, JHA, JSA, JSEA, WMS, TAKE 5, and Incident Investigation. Proceedings of the ASSE Professional Development Conference and Exposition.

[263] Mata O. The Role of Quantitative Risk Analysis QRA in Risk Management. Proceedings of the SPE Latin-American and Caribbean Health, Safety, Environment and Social Responsibility Conference.

[264] Kandil M.E. Efforts to Persevere a Risk Assessment/Integrity Assurance for its Aged Hydrocarbon Transfer Pipe-lines. Proceedings of the SPE Annual Technical Conference and Exhibition.

[265] Cherubin P., Scataglini L., Decarli L., Chisari V., Bandini R. A New Methodology for Major Accident Hazard Risk Assessment According to Eu Directive on Offshore Safety. Proceedings of the Offshore Mediterranean Conference and Exhibition.

[266] Abas N.H., Jalani A.F.A., Affandi H.M. (2020). Construction Stakeholders’ Perceptions of Occupational Safety and Health Risks in Malaysia. Int. J. Sustain. Constr. Eng. Technol..

[267] Mayfield J.A. Process Improvement Based on a Gap Assessment of NASA and O&G Risk Management Processes. Proceedings of the Offshore Technology Conference.

[268] Donaldson B., Shah B., Daher E. Comprehensive Gap Assessment of Complete Safety Management Systems with Systematic Analysis and Recommendations—A Successful Case Study. Proceedings of the SPE Latin American and Caribbean Health, Safety, Environment and Sustainability Conference.

[269] Bendickson N. Don’t Wait for an Intervention--Use CSA “Safety Management Cycle” to Identify Opportunities. Proceedings of the ASSE Professional Development Conference and Exposition.

[270] Diara M., Ngunjiri S., Strohkorb J. Effective Prevention of Tuberculosis Transmission in Oil and Gas Workplaces: A Programmatic Approach. Proceedings of the SPE International Conference and Exhibition on Health, Safety, Security, Environment, and Social Responsibility.

[271] Singhal G., Dibua O., Murray D., Culembourg L., Erb P., Wensel E., Makogon T. Review of Technology Status and Challenges Associated with Ultra Deep Water Developments. Proceedings of the Offshore Technology Conference.

[272] Holtman J.G., Kalb A.T. Enhancement of Corporate Operational Excellence Management System Audit Protocols to Drive Audit Consistency. Proceedings of the SPE International Conference on Health, Safety, and Environment.

[273] Hall J.N. Safety Survey Using Piper Alpha Disaster Evidence. Proceedings of the Offshore Technology Conference.

[274] Luck C.J., Sellers J.G. Safety and Environmental Auditing for Existing Offshore Facilities. Proceedings of the SPE OffShore Europe.

[275] Techasirithaworn M., Tachavarakul V., Krittaphol N., Grassian D. Life Extension of the Jasmine and BanYen Oil Field Exceeding Expectations While Managing Constraints. Proceedings of the Abu Dhabi International Petroleum Exhibition & Conference.

[276] Boutrot J., Giorgiutti Y., Rezende F., Barras S. Reliable and Accurate Determination of Life Extension for Offshore Units. Proceedings of the Offshore Technology Conference.

[277] Morandini C., Floury J. Pragmatic and Consistent Approach to Life Extension of Floating Structures. Proceedings of the Offshore Technology Conference Asia.

[278] Boutrot J., Legregeois N. Integrity Management of Ageing Offshore Assets: An Integrated Approach Towards Life Extension and Operational Efficiency. Proceedings of the SNAME 20th Offshore Symposium.

[279] Hua D.S., Gibbs B. Effective Development of Life Extension Programs for Aging Offshore Facilities. Proceedings of the SNAME 19th Offshore Symposium.

[280] Nashikkar D., Mo W., Achanta V., Lyon S. Riser and Subsea Offshore Asset Field Life Extension. Proceedings of the SNAME 20th Offshore Symposium.

[281] Szalewski P., Malinowski G., Lu J.Y. Demonstrating Target Safety Level for Life Extension of Offshore Structures. Proceedings of the SNAME 24th Offshore Symposium.

[282] Gordon R. Considerations for Mooring Life Extension. Proceedings of the SNAME 20th Offshore Symposium.

[283] Yao A., Zhang Q. Review of Offshore Mooring System Life Extension—Challenges and Recommendations. Proceedings of the SNAME 24th Offshore Symposium.

[284] Forsyth M., Bruno P. Life Extension of Pierce Field Production Facilities, North Sea, UK. Proceedings of the SPE Annual Caspian Technical Conference and Exhibition.

[285] Galbraith D.N., Sharp J.V., Terry E. Managing life extension in Ageing offshore Installations. Proceedings of the SPE Offshore Europe Oil and Gas Exhibition and Conference.

[286] Kajuputra A.E., Shiiun W.B., Shamsuddin M.A. The Importance of SIMS in Structural Integrity Review and Life Extension Requirement for Existing Fixed Offshore Structure. Proceedings of the Offshore Technology Conference Asia.

[287] Westlake H.S., Puskar F.J., O’Connor P.E., Bucknel J.R. The Role of Ultimate Strength Assessments in the Structural Integrity Management (SIM) of Offshore Structures. Proceedings of the Offshore Technology Conference.

[288] O’Connor P.E., Bucknell J.R., DeFrance S.J., Westlake H.S., Westlake F.J. Structural Integrity Management (SIM) of Offshore Facilities. Proceedings of the Offshore Technology Conference.

[289] Westlake H.S., Puskar F.J., O’Connor P.E., Bucknell J.R. The Development of a Recommended Practice for Structural Integrity Management (SIM) of Fixed Offshore Platforms. Proceedings of the Offshore Technology Conference.

[290] Defranco S., O’Connor P., Puskar F., Bucknell J.R., Digre K.A. API RP 2SIM: Recommended Practice for Structural Integrity Management of Fixed Offshore Platforms. Proceedings of the Offshore Technology Conference.

[291] Kongchang A., Danthainum A., Chattratichart T., Sonachai P., Thavornsuk T., Katanyoowongcharoen S., Sriparagul J., Kosanunt P. Alternative Framework for Structural Integrity Management of Jacket Platform Beyond its Design Life. Proceedings of the Abu Dhabi International Petroleum Exhibition & Conference.

[292] Ritchie D.M. The Role of Asset Integrity and Life Extension in Major Accident Prevention. Proceedings of the SPE Offshore Europe Oil and Gas Conference and Exhibition.

[293] Nezamian A., Iqbal K. Requalification and Extension of Service Life and Integrity Requirements for Offshore Structures in Middle East. Proceedings of the International Petroleum Technology Conference.

[294] Fairbairn L., Pegram A., Nishapati M. E-Safety Case—Improving Communication and Application of HSE Data. Proceedings of the SPE International Health, Safety & Environment Conference.

[295] Scanlon M. Revised Industry Guidance on Managing Safety Critical Elements. Proceedings of the Abu Dhabi International Petroleum Exhibition & Conference.

[296] Bentley N.L., Seaman C.H., Brower D.V., Tang H.H., Le S.Q. (2017). Development and Testing of a Friction-Based Post-Installable Fiber-Optic Monitoring System for Subsea Applications. Proceedings of the ASME 2017 36th International Conference on Ocean, Offshore and Arctic Engineering.

[297] Cortina M., Pavli E., Antinolfi G., La Rosa L., Rainaldi I., Petrone A. An Overarching Strategy for Safety Critical Elements Assessment and Management. Proceedings of the SPE International Conference on Health, Safety, and Environment.

[298] Bhat S. Safety-Critical Elements/Performance Standards—Engineering. LinkedIn Pulse, 8 June 2021. https://www.linkedin.com/pulse/safety-critical-elementsperformance-standards-engineering-bhat?trk=public_profile_article_view.

[299] Dhar R. Performance Standards for Safety Critical Elements—Are We Doing Enough?. Proceedings of the SPE European Health, Safety and Environmental Conference in Oil and Gas Exploration and Production.

[300] Denney D. (2011). Performance Standards for Safety-Critical Elements—Are We Doing Enough?. J. Pet. Technol..

[301] Pillai S. Best Practices in Integrity Management of Safety Critical Systems. Proceedings of the Abu Dhabi International Petroleum Exhibition & Conference.

[302] Wilson A. (2015). Safety Case in the Gulf of Mexico: Method and Benefits for Old and New Facilities. J. Pet. Technol..

[303] McIntosh A.M. Features of a Safety Case for a Complex of Platforms with NNM’s. Proceedings of the SPE Offshore Europe.

[304] Hart P.M.I., Smith D.W., Thomas E.J. Preparation of Combined Operations Safety Cases: Experience and Lessons Learnt. Proceedings of the SPE Offshore Europe.

[305] Whewell I. Safety Cases—10 Years of Experience. Proceedings of the SPE International Conference on Health, Safety, and Environment in Oil and Gas Exploration and Production.

[306] Spittal J. The Ivanhoe/Rob Roy Safety Case Development. Proceedings of the SPE Offshore Europe.

[307] Stiff J., Cusano D., Taylor E. The Safety Case: Where it Came from and where it is Going. Proceedings of the SNAME 18th Offshore Symposium.

[308] Carval J., Das B. Safety Case in GoM: Method and Benefits for Old and New Facilities. Proceedings of the Offshore Technology Conference.

[309] Alme I., Wingate K., Dunn C. Safety Case for Gulf of Mexico—What Would It Mean? A Global Case Study of the Application of a Safety Case Regime. Proceedings of the Offshore Technology Conference.

[310] Wong N. Piloting Safety Cases to Support Decisive Management of Change. Proceedings of the SPE Offshore Europe.

[311] Bennett R., Ramsden M., Steer R. Maximizing Safety Case Value with Meaningful Workforce Engagement. Proceedings of the SPE International Conference on Health, Safety, and Environment.

[312] Lo Brutto F.M., Layfield M. The New EU Offshore Safety Directive—Key Requirements and Impacts. Proceedings of the Offshore Mediterranean Conference and Exhibition.

[313] Uguccioni G., Rentocchini P., Giacchino C. EU Directive on Offshore Safety—Problem or Opportunity?. Proceedings of the Offshore Mediterranean Conference and Exhibition.

[314] Pat-El I.E., Meijlink L., Mol P. Major Accidents and their impact—The EU Directive for Offshore Safety. Proceedings of the Abu Dhabi International Petroleum Exhibition and Conference.

[315] Viramuthu V.A. Human Factors Engineering HFE: A Review of Human Elements that have Contributed in the Occurrence of Major Accidents in Process Industries and Total Abu Al Bukhoosh TABK Means of Engineering Effective Mitigations. Proceedings of the Abu Dhabi International Petroleum Exhibition & Conference.

[316] Buus L., Covarrubias E., Lyager K., Beks R. Systematic Risk Based Verification of Barriers—While Implementing the EU Offshore Safety Directive. Proceedings of the Offshore Mediterranean Conference and Exhibition.

[317] Derevyakin M. Systematic Approach Ensuring Asset Integrity, Equipment Reliability and Process Safety. Proceedings of the SPE Russian Oil and Gas Conference and Exhibition.

[318] Kamal G. Assurance and Verification of Safety Critical Elements in Asset Management. Proceedings of the International Petroleum Technology Conference.

[319] Marty J., Theys S.O.P., Bucherie C., Bolsover A., Cambos P. Independent Verification of Safety Critical Elements. Proceedings of the SPE Russian Oil and Gas Conference and Exhibition.

[320] Powell T., Dobson J., Dykes C. Changes to Regulation of Drilling and Workovers in UK Sector by Removal of Prescription. Proceedings of the SPE Offshore Europe.

[321] Romagnoli R., Bosio E. Evolution of the Drilling Mud Pumping Systems: Related Safety Standards and Actual Risk Analysis Upgrades in Offshore. Proceedings of the Thirteenth International Offshore and Polar Engineering Conference.

[322] Royle D.J.C. Workforce Involvement in the UK Offshore Oil & Gas Industry. Proceedings of the SPE Offshore Europe.

[323] Rose D., Crescent J. Evaluation of the Offshore Safety Legislative Regime in the U.K. Proceedings of the SPE International Conference on Health, Safety and Environment in Oil and Gas Exploration and Production.

[324] Perham A.J., Garlick A.R., Forster J.H. Goal-Setting Regulation: Truly Cost-Effective Safety?. Proceedings of the SPE Offshore Europe.

[325] Taylor B.G.S. A Goal-Setting Approach to Offshore Regulations. Proceedings of the SPE Offshore Europe.

[326] Sprague C.W. The New Offshore Health and Safety Regulations: A Legal Overview. Proceedings of the SPE Offshore Europe.

[327] Norman P., Lochte G., Hurley S. White Rose: Overview of current development and plans for future growth. Proceedings of the 18th International Offshore and Polar Engineering Conference.

[328] Pardy C., Akinniranye G., Carter M., Crane G., Wishart L., Krepp T., Foster B. White Rose project drilling and completion performance evolution: A case study. Proceedings of the SPE/IADC Drilling Conference and Exhibition.

[329] Anvik H.K., Gibson W.R. Drilling and Workover Experiences in the Greater Ekofisk Area. Proceedings of the SPE/IADC Drilling Conference.

[330] Bickley M.C., Curry W.E. Designing Wells for Subsidence in the Greater Ekofisk Area. Proceedings of the European Petroleum Conference.

[331] Dechant S., McFadyen M.K. Kikeh Development: Project Execution Model. Proceedings of the Offshore Technology Conference.

[332] MdSalleh N.B., Sainal M.R.B. Malaysia Deepwater Project Execution Strategy and Challenges. Proceedings of the Abu Dhabi International Petroleum Exhibition and Conference.

[333] Hassani V., Pascoal A.M., Sørensen A.J. (2018). Detection of mooring line failures using dynamic hypothesis testing. Ocean Eng..

[334] Siréta F., Zhang D. Smart mooring monitoring system for line break detection from motion sensors. Proceedings of the 13th ISOPE Pacific/Asia Offshore Mechanics Symposium.

[335] Liu Y., Fontanella A., Wu P., Ferrari R., Wingerden J. (2020). Fault detection of the mooring system in floating offshore wind turbines based on the wave-excited linear model. J. Phys. Conf. Ser..

[336] Dan S. (2006). Optima State Estimation.

[337] Ayaz E. (2015). Detection and identification of mechanical faults by Kalman filtering in electric machines. J. Vibroeng..

[338] Eykeren L.V., Chu Q., Mulder J. (2012). Sensor fault detection and isolation using adaptive extended Kalman filter. IFAC Proc..

[339] Jesussek M., Ellermann K. (2014). Fault detection and isolation for a full-scale railway vehicle suspension with multiple kalman filters. Veh. Syst. Dyn..

[340] Auger F., Hilairet M., Guerrero J., Monmasson E., Orlowska-Kowalska T., Katsura S. (2013). Industrial applications of the Kalman filter: A review. IEEE Trans. Ind. Electron..

[341] Beltran J., Williamson E. (2011). Numerical procedure for the analysis of damaged polyester ropes. Eng. Struct..

[342] Imai H., Yun C.B., Maruyama O., Shinozuka M. (1989). Fundamentals of system identification in structural dynamics. Probab. Eng. Mech..

[343] Zhao J., Su Y. EKF moving horizon estimation based nonlinear filter for marine dynamic positioning system. Proceedings of the 2015 Chinese Automation Congress (CAC).

[344] Perez T.F.T. (2010). Kalman filtering for positioning and heading control of ships and offshore rigs. CST.

[345] Triantafyllou M., Bodson M., Athans M. (1983). Real time estimation of ship motions using Kalman filtering techniques. IEEE J. Ocean. Eng..

[346] Alcocer A., Oliveira P., Pascoal A. (2007). Study and implementation of an EKF gib-based underwater positioning system. Control Eng. Pract..

[347] Grimble M., Patton R.J., Wise D.A. (1980). Use of Kalman filtering techniques in dynamic ship-positioning systems. Proc. IEEE.

[348] Balchen J.G., Jenssen N.A., Mathisen E., Saelid S. (1980). Dynamic positioning of floating vessels based on Kalman filtering and optimal control. Proceedings of the 19th IEEE Conference on Decision and Control including the Symposium on Adaptive Processes.

[349] Tockner A., Blümel B., Ellermann K. (2021). Fault Detection in Modular Offshore Platform Connections Using Extended Kalman Filter. Front. Built Environ..

[350] Newman J. (1977). Marine Hydrodynamics.

[351] Foster G.P. (2002). Advantages of Fiber Rope Over Wire Rope. J. Ind. Text..

[352] Oland E., Schlanbusch R., Falconer S. (2017). Condition Monitoring Technologies for Synthetic Fiber Ropes—A Review. Int. J. Progn. Heal. Manag..

[353] Gordelier T., Thies P.R., Rinaldi G., Johanning L. (2020). Investigating Polymer Fibre Optics for Condition Monitoring of Synthetic Mooring Lines. J. Mar. Sci. Eng..

[354] Garrido R., Rivero-Angeles F.J., Martinez-Guerra R., Gomez-Gonzalez B., Martinez-Garcia J.C. Nonlinear Restoring Force Estimation in Civil Structures Using a High Gain Observer. Proceedings of the 5th Asian Control Conference (IEEE Cat. No.04EX904).

[355] Lin J.-W., Betti R. (2004). On-line identification and damage detection in non-linear structural systems using a variable forgetting factor approach. Earthq. Eng. Struct. Dyn..

[356] Farza M., Saad M.M., Maatoug T., Kamoun M. (2009). Adaptive observers for nonlinearly parameterized class of nonlinear systems. Automatica.

[357] Torres L., Verde C., Hernandez A.O.V. (2015). Parameter identification of marine risers using Kalman-like observers. Ocean Eng..

[358] Mu H.-Q., Kuok S.-C., Yuen K.-V. (2017). Stable Robust Extended Kalman Filter. J. Aerosp. Eng..

[359] Koh C.G., See L.M., Balendra T. (1991). Estimation of structural parameters in time domain: A substructure approach. Earthq. Eng. Struct. Dyn..

[360] Tockner A., Lei J., Ellermann K. (2022). Fault Detection in Offshore Structures: Influence of Sensor Number, Placement and Quality. Appl. Mech..

[361] Yang Q., Li J., Santos R., Huang K., Igic P. (2020). Intelligent fault detection and location scheme for modular multi-level converter multi-terminal high-voltage direct current. High Volt..

[362] Liu Y., Ferrari R., Wu P., Jiang X., Li S., van Wingerden J.-W. (2021). Fault diagnosis of the 10MW Floating Offshore Wind Turbine Benchmark: A mixed model and signal-based approach. Renew. Energy.

[363] Xu X., Yan X., Yang K., Zhao J., Sheng C., Yuan C. (2021). Review of condition monitoring and fault diagnosis for marine power systems. Transp. Saf. Environ..

[364] Zhu J. (2021). Review on Structural Health Monitoring of Offshore Platform. J. Phys. Conf. Ser..

[365] Yanlin W., Xiangjun B., Sheng F., Yingxin M., Qianjin Y. (2011). Subsidence monitoring of offshore platforms. Procedia Eng..

[366] Aqdam H.R., Ettefagh M.M., Hassannejad R. (2018). Health monitoring of mooring lines in floating structures using artificial neural networks. Ocean Eng..

[367] Wang P., Tian X., Peng T., Luo Y. (2018). A review of the state-of-the-art developments in the field monitoring of offshore structures. Ocean Eng..

[368] Peng R., Zhi Z. (2012). A state-of-the-art review on structural health monitoring of deepwater floating platform. Pac. Sci. Rev..

[369] Gordon R., Brown M., Allen E. Mooring Integrity Management: A State-of-the-Art Review. Proceedings of the Offshore Technology Conference.

[370] Chan P.H. (2015). Design Study of Composite Repair System for Offshore Riser Applications. Ph.D. Thesis.

[371] Alexander C.R. (2007). Development of Composite Repair System for Reinforcing Offshore Risers. Ph.D. Thesis.

[372] Alexander C., Ochoa O.O. (2010). Extending onshore pipeline repair to offshore steel risers with carbon–fiber reinforced composites. Compos. Struct..

[373] Ochoa O.O., Alexander C. Hybrid Composite Repair for Offshore Risers. Proceedings of the 17th International Conference of Composite Materials (ICCM17).

[374] Jacques R.C., Flores J.V., Strohaecker T.R., Reguly A. (2009). Acoustic emission testing in wires from the tensile armour of flexible risers under load. Insight—Non-Destr. Test Cond. Monit..

[375] Alexander C., Brooks C. Development and Evaluation of a Steel-Composite Hybrid Composite Repair System. Proceedings of the 9th International Pipeline Conference (IPC 2012).

[376] Alexander C. Advanced Techniques for Establishing Long-Term Performance of Composite Repair Systems. Proceedings of the 10th International Pipeline Conference (IPC 2014).

[377] Alexander C., LaVergne R., Turner A. Use of Fiber Optic Technology in Monitoring Steel Sleeves and Composite Wrap Reinforcements. Proceedings of the 12th International Pipeline Conference (IPC 2018).

[378] Elosta H., Gavouyere T., Garnier P. Flexible Risers Lifetime Extension: Riser In-Service Monitoring and Advanced Analysis Techniques. Proceedings of the ASME 2017 36th International Conference on Ocean, Offshore and Arctic Engineering.

[379] Criado A., Riezu M., Fernandez A., Oizm A. Evaluation of OBR for Strain Measurements in Blade Testing. Proceedings of the European Wind Energy Conference.

[380] Díaz-Maroto P., López A.F., Larrañaga B., Gordo J.A.G. Free-Edge Delamination Location and Growth Monitoring with an Embedded Distributed Fiber Optic Network. Proceedings of the 8th European Workshop on Structural Health Monitoring (EWSHM 2016).

[381] Inaudi D., Glisic B., Gasparoni F., Cenedese S., Zecchin M. Strain Sensors for Deepwater Applications. Proceedings of the 3rd International Conference on Structural Health Monitoring of Intelligent Infrastructure.

[382] Cook H., Dopjera D., Thethi R., Williams L. Riser Integrity Management for Deepwater Developments. Proceedings of the Offshore Technology Conference.

[383] Thethi R., An P. Performance Monitoring of Deepwater Risers. In Proceeding of the 27th International Conference on Offshore Mechanics and Arctic Engineering.

[384] Thethi R., Howells H., Natarajan S., Bridge C. A Fatigue Monitoring Strategy and Implementation on a Deepwater Top Tensioned Riser. Proceedings of the Offshore Technology Conference.

[385] Morrison D., Dean J. (2007). Apparatuses and Methods for Monitoring Stress in Steel Catenary Risers. U.S. Patent.

[386] Allen J., Pinto A. (2008). System for Sensing Riser Motion. U.S. Patent.

[387] Alexander C., Vyvial B., Cederberg C., Baldwin D. Evaluating the Performance of a Composite-Reinforced Steel Drilling Riser via Full-Scale Testing for HPHT Service. Proceedings of the 6th International Offshore Pipeline Forum (IOPF 2011).

[388] Jacques R., Clarke T., Morikawa S., Strohaecker T. (2010). Monitoring the structural integrity of a flexible riser during dynamic loading with a combination of non-destructive testing methods. NDT E Int..

[389] Prescott C., Brower D. (2005). Real Time Subsea Monitoring and Control System for Pipelines. U.S. Patent.

[390] Williams J. (2007). Dynamic Performance Monitoring of Long Slender Structures Using Optical Fiber Strain Sensors. U.S. Patent.

[391] Brower D., Hedengren J.D., Shishivan R.A., Brower A. Advanced Deepwater Monitoring System. In Proceeding of the 32nd International Conference on Ocean, Offshore and Arctic Engineering.

[392] Offshore Technology (2022). ROSEN Group—Integrity Management for Offshore Assets.

[393] Dodds D.J. (1999). Anchor Monitoring System. U.S. Patent.

[394] Schneider L. (1971). Anchor Dragging Alarm Control Based on Anchor Inclination. U.S. Patent.

[395] (1973). Global Marine Inc. Load Indicator for Mooring Line. U.S. Patent.

[396] (1975). Secretary Trade Ind Brit. Monitoring System for Moored Floating Vessels. U.S. Patent.

[397] (1981). Decker Engineering Corp. Load Indicating Apparatus Having a Hydraulic Sensing Unit and Coupling Pin Type Electronic Sensing Unit. U.S. Patent.

[398] Oettli M.W. (1987). Method for Monitoring the Drift of an Anchored Vessel and Device for Implementing the Method. U.S. Patent.

[399] Douglas H.S. (2004). Load Monitoring Systems and Methods. U.S. Patent.

[400] Vetco A.A. (2007). Method and a Device for Monitoring an/or Controlling a Load on a Tensioned Elongated Element. U.S. Patent.

[401] Michie B.E. (2009). Method of Determining and Monitoring a Distance Travelled by a Marine Vessel Connected to Anchor. U.S. Patent.

[402] (2010). Hifunda, Llc. Method and Device for Harvesting Energy from Ocean Waves. U.S. Patent.

[403] Prentice W.M. (2013). Monitoring of Underwater Mooring Lines. U.S. Patent.

[404] (2018). Fujitsu Limited. Alert Control System, Alert Control Method, and Recording Medium Storing Alert Control Program. U.S. Patent.

[405] Bermudez R.J. (2003). Method of Monitoring/Measuring Rigging Loads. U.S. Patent.

[406] Cheung M.C. (2005). Tension Measuring Device for Mooring Line. U.S. Patent.

[407] Hyland B.P. (2021). Subsea Structure Monitoring System. U.S. Patent.

[408] General Electric Co. (2016). System and Method for Monitoring Tubular Components of a Subsea Structure. U.S. Patent.

[409] General Electric Co. (2019). Marine Riser Management System Including Subsea Acoustic Monitoring Platform and an Associated Method. U.S. Patent.

[410] Nkt Hv Cables Ab. (2022). Method and System for Fatigue-Monitoring of a Submarine Cable in Off-Shore Operations. U.S. Patent.

[411] Salama M. (2015). Composite Riser with Integrity Monitoring Apparatus and Method. U.S. Patent.

